# Data-Minimizing Motion-Gated Artificial Intelligence Decision Support for Electric Micro-Mobility: An Architecture-Feasibility Study

**DOI:** 10.3390/s26196334

**Published:** 2026-10-07

**Authors:** Syed Tahir Ali Shah, José Paulo Oliveira Santos, José Maria Fernandes, António B. Pereira, Gabriel Constantinescu

**Affiliations:** 1 TEMA—Centre for Mechanical Technology and Automation, Department of Mechanical Engineering, University of Aveiro, Campus Universitário de Santiago, 3810-193 Aveiro, Portugal; jps@ua.pt (J.P.O.S.); abastos@ua.pt (A.B.P.); gabriel.constantinescu@ua.pt (G.C.); 2 IEETA—Institute of Electronics and Informatics Engineering of Aveiro, Department of Electronics, Telecommunications and Informatics, University of Aveiro, Campus Universitário de Santiago, 3810-193 Aveiro, Portugal; jfernan@ua.pt

**Keywords:** artificial intelligence, decision support, data minimization, motion-reliability gating, exertion-aware classification, electric micro-mobility, architecture-feasibility study, e-scooter, e-bike, wearable sensing

## Abstract

Physiological sensing can support electric micro-mobility safety research. Elevated heart rate during riding, however, may reflect physical exertion, psychological stress, motion artefacts, road context, or several of these factors. This paper presents RideSafe-AI, a non-clinical architecture-feasibility prototype. It separates data quality, exertion/load proxy estimation, public-dataset stress-label classification, and motion reliability before applying deterministic fusion and abstention. The Python reference checks input and output fields for raw physiological streams and direct identifiers, while the ESP32-S3 (Espressif Systems, Shanghai, China) checks incoming fields; Android constructs bounded inputs internally without an equivalent schema guard. PAMAP2 supports exertion/load proxy modelling, WESAD supports low/high laboratory condition-label classification, and official PhysioNet stress/exercise records support a subject-wise laboratory feature-set experiment. On the PhysioNet binary stress-versus-exercise task, full-feature XGBoost achieved 0.963 accuracy, 0.944 macro-F1, and a 0.015 exercise false-stress rate on 10,824 windows from 36 subjects. These results describe that public-dataset task; PhysioNet does not contain bike-frame motion, GPS terrain, or power telemetry and does not evaluate the complete RideSafe-AI pipeline. Deterministic scenarios verify selected decision rules, while two same-day technical rides by one participant demonstrate prototype integration and logging. These findings support architecture feasibility. Rider-stress accuracy, safety impact, and performance across riders and road conditions remain to be evaluated.

## 1. Introduction

Electric micro-mobility systems—e-bikes and e-scooters—increasingly rely on connected sensors, wearable devices, and edge computing to support safety-oriented user awareness and research. Rider-state monitoring is a safety-relevant problem in this context: prior work links route infrastructure to cyclist injury risk and shows that e-bike use, mode choice, and e-cyclist behavior differ measurably from conventional cycling [1,2,3,4,5]. Physiological signals such as heart rate can provide useful information about rider state, and prior driving-stress and wearable-stress work has shown that physiological signals can be informative in transport and daily-life settings [6,7,8,9]. However, interpreting these signals during riding is not straightforward. A high heart rate may be caused by physical exertion, environmental load, psychological stress, road slope, or a mixture of these factors. A rider-facing system that treats all physiological elevation as stress risks generating misleading alerts and increasing distraction.

A second challenge is motion. Wrist-worn optical sensing is commonly based on photoplethysmography (PPG), and PPG-derived heart-rate estimates are affected by sensor placement, contact quality, activity, and motion artefacts [10,11,12]. Bike-mounted inertial sensors can help characterize vehicle-frame motion, but they do not directly measure psychological stress. A reliable rider-state system therefore needs to separate at least four concerns: physical exertion, public-dataset stress-label evidence, motion reliability, and decision confidence.

A third challenge is privacy. Physiological streams may be sensitive data, and health-related information is regulated as a special category of personal data under General Data Protection Regulation (GDPR) Article 9 [13]. A rider-state artificial intelligence system should not need unrestricted access to raw physiological streams in order to produce safety recommendations. Instead, raw or near-raw signals should be transformed into limited feature summaries before final decision logic.

This paper introduces RideSafe-AI, a bounded decision-support architecture for rider-context advisory support. In this manuscript, the architecture is the organization of processing stages and interfaces. The prototype is the implemented realization across Python, Android, and ESP32-S3 tiers. The decision-support system is the overall advisory function. The term agent refers to the bounded software pipeline that applies declared processing steps and deterministic fusion rules to bounded feature windows; it is not a general-purpose autonomous assistant or a clinical diagnostic agent. RideSafe-AI treats elevated physiology as ambiguous until data quality, exertion/load evidence, public-dataset stress-label evidence, and motion reliability have been evaluated separately. Its purpose in this study is to examine architecture feasibility and conservative advisory behavior under the stated tests.

Here, rider context denotes recorded physiological, motion, terrain, and power information. In descriptions of the prototype, rider state denotes an intermediate categorical assessment produced by the decision rules. It is not an independently verified psychological state. Stress-label classification denotes prediction of the labels defined by a public dataset, and stress-label evidence denotes that branch’s output.

The current prototype also extends physiology-only inference with ride-context logging. It records GPS terrain, motion-reliability, and power-telemetry fields for later tests of whether riding context changes the interpretation of elevated heart rate (see Materials and Methods for the full bounded input schema). The current results demonstrate logging and fusion feasibility; they do not complete rider-context validation.

The rider-facing nature of the application is also informed by human–automation literature, which motivates conservative advisory outputs, transparency, and appropriate reliance rather than opaque automation [14,15,16,17,18,19].

### 1.1. Research Gap

Existing wearable stress pipelines and activity-recognition datasets provide useful modelling evidence, but an electric micro-mobility context introduces a specific ambiguity: physical exertion and psychological stress can both raise physiological load. Wearable-only disambiguation of physical exertion and psychological stress remains an open problem. In a six-participant laboratory study, a five-condition task covering rest, physical stress, physical recovery, psychological stress, and psychological recovery achieved 77.8% overall accuracy using heart rate, heart-rate variability, electrodermal activity, and accelerometry; adding salivary cortisol features increased overall accuracy to 94.4% [20]. A related multi-task pipeline jointly classifies physical activity and acute psychological stress from wearable data without a motion-reliability or data-minimizing output boundary [21]. Neither study evaluates electric micro-mobility or reports feasibility on constrained edge hardware. A rider-state system also has to decide whether motion artefacts make a prediction reliable enough to expose to a rider. The research gap addressed here is therefore not simply stress classification. The gap is data-minimizing, motion-gated, exertion-aware decision support for electric micro-mobility rider-context monitoring.

### 1.2. Research Questions

The study is organized around six research questions. RQ1 and RQ2 form the primary research question, testing whether the architecture can separate decision evidence into traceable steps and whether adding exertion-related information measurably reduces exercise-induced false-stress classification in public stress/exercise data; RQ3–RQ6 test supporting mechanisms within that same architecture.

1. RQ1: Can the decision pipeline separate exertion/load evidence, public-dataset stress-label evidence, motion reliability, and data quality into traceable steps for rider-context advisory support?
2. RQ2: Under subject-independent public-data evaluation, does adding exertion-related physiological and motion information reduce exercise-induced false stress classification relative to a stress-focused baseline, and does the full RideSafe-AI agent preserve this behavior in designed rider-state scenarios?
3. RQ3: Can the final decision-stage interface enforce a privacy boundary by rejecting raw physiological streams and direct identifiers?
4. RQ4: Is it feasible to implement the RideSafe-AI decision pipeline on an Android phone and an ESP32-S3 microcontroller while retaining a Python reference for comparison and reporting?
5. RQ5: Can GPS terrain context and power telemetry be logged in the bounded schema needed to identify candidate false-stress escalations for later validation?
6. RQ6: Can comparison of the Android and ESP32-S3 outputs expose implementation or motion-gate mismatches during technical ride logging?

### 1.3. Contributions

This paper makes the following contributions. The central contribution is architectural rather than a new classification method: RideSafe-AI keeps exertion/load proxy evidence, public-dataset stress-label evidence, motion reliability, and privacy as separately traceable decision steps, with prototype implementations across three tiers (Python reference, Android phone, and ESP32-S3 microcontroller).

1. Bounded, traceable decision-support architecture. A data-minimizing, motion-gated artificial intelligence decision-support pipeline that separates data quality, exertion/load proxy estimation, public-dataset stress-label classification, motion reliability, advisory-state generation, confidence, abstention, and privacy guarding instead of collapsing them into a single opaque stress classifier.
2. Cross-tier deployment evidence. A prototype split in which wearable and phone streams provide bounded physiological summaries, an ESP32-S3 embedded C++ agent executes privacy guarding, data-quality checks, motion reliability, conservative fusion, and advisory-state generation from aggregate inputs, and a phone-versus-ESP32 comparison exposes agreement and mismatch patterns.
3. Public-data and external evidence base. A dual-branch modelling pipeline that uses PAMAP2 for physical exertion/load proxy modelling [22] and WESAD for low/high public-dataset stress-label classification [23], extended by a protocol-reconstructed, subject-independent PhysioNet stress-versus-exercise feature-set experiment [24,25] with participant-level false-stress-rate statistics across kNN, RF, linear SVM, and XGBoost.
4. Context-aware technical logging. A ride-logging layer that couples smartwatch physiology, e-bike/e-scooter frame IMU features, GPS terrain, and INA219(Texas Instruments, Dallas, TX, USA) power telemetry with self-labelling segment fields for later rider-context validation, currently demonstrated only as single-participant technical feasibility evidence pending recruited-rider validation.

The current study evaluates RideSafe-AI as an architecture-feasibility prototype using public datasets, designed scenarios, robustness tests, edge-device feasibility checks, and two same-day technical ride logs from a single participant. Broader rider-context and safety-impact claims are left to future ethics/GDPR-approved validation studies. The study follows a design-science-based pre-deployment evaluation framework [26]. System- and model-level reporting uses DECIDE-AI- and TRIPOD+AI-inspired principles as non-clinical reporting scaffolds rather than clinical validation guidelines [27,28]. The Architecture and Materials and Methods sections describe this framework in detail.

## 2. Related Work

### 2.1. Wearable Stress and Transport Context

Physiological stress detection has been studied in transport settings, including real-world driving tasks using physiological sensors [6]. Broader wearable-stress literature also shows that phone and wearable signals can support stress inference, while emphasizing that daily-life deployment is complicated by context, activity, person-specific variation, and label quality [7,8,9,29,30,31,32]. WESAD is a multimodal wearable dataset for stress and affect detection. It includes physiological signals such as wrist acceleration, BVP, EDA, and temperature, and provides condition labels that can be used for low/high stress modelling [23]. In RideSafe-AI, WESAD is used only for public stress-label model development. This is a useful but limited source of evidence because WESAD is not an electric micro-mobility traffic dataset. The specific problem of separating physical and psychological stress from wearable signals alone has been directly targeted by other groups: a laboratory study found that wearable-only features (heart rate, heart-rate variability, electrodermal activity, and accelerometry) classify physical versus psychological stress and recovery states with 77.8% accuracy, rising to 94.4% only when salivary cortisol samples are added [20], while a multi-task wearable pipeline jointly classifies physical activity and acute psychological stress from Empatica E4 (Empatica Inc., Boston, MA, USA) wristband blood-volume-pulse, skin-temperature, electrodermal-activity, and acceleration signals [21]. Both results indicate that wearable-only physical/psychological stress disambiguation is a hard, still-open problem rather than a solved one; RideSafe-AI does not attempt to outperform these accuracy figures, and instead targets a real-time, sensor-only, edge-deployable operating point that neither approach evaluates. Complementary ambulatory-stress work shows that accelerometer-derived posture and activity information can be used to adjust heart rate for physical-activity confounds before assessing mental-stress reactivity, though the resulting adjusted signal still only weakly separates physical from mental demands on its own [33].

### 2.2. Physical Activity and Exertion Context

PAMAP2 is a physical activity monitoring dataset that includes heart-rate and inertial data from multiple activities, including cycling [22,34]. In RideSafe-AI, PAMAP2 is used for exertion/load proxy modelling. It is not used as a psychological stress dataset because it does not provide stress labels. This separation is central to the proposed architecture.

A newer PhysioNet wearable dataset directly strengthens this separation because it contains Empatica E4 physiological signals collected during both induced stress tasks and structured aerobic/anaerobic exercise sessions, including stationary-bike exercise [24]. The accompanying Scientific Data article documents the recording protocol, the 36-participant cohort, and the authors’ own machine learning benchmark for stress-versus-rest, aerobic-versus-anaerobic, and four-class classification [25]; Section 6.5 compares that published benchmark with the current LOSO results and states the validation-protocol differences explicitly rather than implying superiority. The dataset is therefore a better public bridge between WESAD-style stress labels and PAMAP2-style exertion/load evidence, although it is still not an on-road micro-mobility dataset. The extended reproducibility audit reprocesses the official PhysioNet protocol folders and event tags rather than relying on the older all-stress derived support file; the resulting labels are used only for public stress/rest/exercise protocol-window checks, not as rider traffic validation, and this feature-set-level PhysioNet experiment is kept terminologically distinct from the RideSafe-AI agent ablation (Section 5.3). For ride-context support, recent cycling-stress work combines wearable electrodermal-activity measurements with geo-referenced trip data and open spatial data (OpenStreetMap, Sentinel-2, and street-view imagery) across 1780 cycling trips by 89 participants [35], while e-bike and micromobility-specific data sources provide complementary ride context: the GiroE-2024 e-bike smartwatch dataset reports GNSS, heart-rate, altimeter, accelerometer, compass, and temperature data from e-bike stages, without stress labels [36], and a naturalistic instrumented-e-bike study separately reports chest-worn heart rate together with a peripheral-detection mental-workload measure across real urban routes, finding that workload and heart rate rise at lower speeds in dense traffic [37]. Recent e-scooter/bicycle-lane studies report EDA-based physiological stress and riding-behavior variables in controlled virtual-reality micromobility scenarios [38], and a related virtual-reality comparison from the same research group finds that e-scooter riders show lower physiological stress than bicycle riders under matched shared-lane conditions, with age-dependent effects [39]. A related companion paper by the present author team reports a separate five-trial e-scooter HR/GPS traffic-density study in Oliveira do Bairro, Portugal [40]; that work provides real-route context evidence for traffic-associated heart-rate variation, whereas the present manuscript contributes the decision architecture, data-minimizing guard, motion gate, PhysioNet subject-wise feature-set analysis, and Android/ESP32 implementation. A complementary naturalistic e-scooter study uses eye-tracking, video, and speed data, rather than physiological signals, to characterize rider gaze behavior and traffic conflicts across pedestrian-shared paths, cycle lanes, and roadways [41]; this behavioral/traffic-conflict evidence is a different measurement axis from the physiological rider-state evidence RideSafe-AI targets, but it reinforces that e-scooter-specific riding contexts differ measurably from cycling. These sources do not replace recruited RideSafe-AI rider validation, but they define a stronger external-data path for future model transfer and protocol design. A companion PRISMA-guided systematic review by the present authors situates these individual sources within the broader micro-mobility sensing landscape: across cycling and e-scooter physiological-safety studies published between 2015 and 2025, laboratory exertion-prediction models report high accuracy but rely on a single, non-independently validated dataset, field studies report only statistical associations between heart-rate variability and environmental stress without real-time predictive capability, and automated physiological safety classification for e-scooters remains critically underdeveloped because cycling-trained models are overfitted to cycling biomechanics and do not account for e-scooter-specific whole-body vibration [42]. That review calls explicitly for unsupervised domain adaptation and noise-resilient edge AI architectures to close this gap; the motion-reliability gating and ESP32-based edge deployment developed here are an initial engineering response to that need; the embedded motion gate is not yet field-calibrated (Section 6.12).

### 2.3. Wearable Activity Recognition

Wearable activity recognition provides the modelling background for interpreting inertial and physiological context. Survey and tutorial work on wearable human activity recognition emphasizes feature-window design, sensor placement, evaluation protocol, and the difficulty of generalization across people and contexts [43,44]. Empirical and benchmark work further shows the importance of windowing, sensor placement, benchmark datasets, and subject-wise evaluation in activity recognition [45,46,47,48,49]. Deep-learning surveys for sensor-based activity recognition further show the potential of learned temporal representations, while also highlighting the need for sufficient labelled data and careful evaluation [50]. RideSafe-AI uses this background to motivate bounded feature windows and explicit separation between activity/exertion evidence and stress evidence.

### 2.4. Wearable Signal Quality and Motion Artefacts

Wearable PPG is a practical basis for heart-rate monitoring, but it is not a motion-free physiological channel. Foundational PPG work describes the optical measurement principle and its physiological interpretation limits [10,51], while wearable PPG reviews describe the shift from clinical pulse oximetry to mobile and wrist-worn monitoring [11,52,53]. Empirical wearable-accuracy work further shows that activity condition and device type can affect optical heart-rate error [12,54,55,56]. Signal processing work for PPG under motion also motivates explicit artefact handling rather than direct trust in every optical heart-rate window [57,58]. Motion reliability in RideSafe-AI is not limited to wrist PPG: a low-cost data-acquisition system with an inertial measurement unit mounted on an e-scooter recorded vertical accelerations that differed markedly between asphalt, concrete, and cobblestone surfaces, indicating that vehicle-frame motion is itself a measurable and relevant signal-quality factor [59]. RideSafe-AI therefore treats motion reliability and data quality as first-class decision inputs rather than assuming that every elevated heart-rate window is reliable stress evidence.

### 2.5. TinyML, Edge AI, and Benchmarking

TinyML enables machine learning inference on microcontrollers and constrained embedded devices. TensorFlow Lite Micro is one representative framework for embedded machine learning in TinyML systems [60], and recent TinyML reviews emphasize tool-chain constraints, reliability, and resource-aware deployment on low-power embedded platforms [61]. A directly relevant precedent already demonstrates TinyML PPG-based stress detection on constrained wearable hardware [62], and a closely related deployment fits a multimodal (accelerometer, temperature, heart rate, and electrodermal activity) XGBoost stress classifier onto a resource-constrained microcontroller [63]; RideSafe-AI’s edge contribution is therefore not the general feasibility of on-device wearable stress inference, but the specific combination of motion-reliability gating, multi-tier phone/microcontroller comparison, and privacy guarding within an edge-deployable pipeline. IoT, edge-computing, and edge-AI work motivate pushing time-sensitive sensing, filtering, and decision support closer to the device rather than relying only on cloud processing [64,65,66,67]. Broader inference benchmarking and efficient-deep-learning work have also emphasized reproducible latency, memory, and deployment measurements rather than model accuracy alone [68,69]. RideSafe-AI uses ESP32-S3 for bike-mounted motion feature extraction, HTTP gateway support, and an embedded C++ version of the decision pipeline. The claim is not that Python, LLM reasoning, or large-model orchestration runs on the microcontroller; rather, the privacy, quality, scoring, motion-gating, fusion, uncertainty, abstention, and safety-state logic is migrated into a compact firmware implementation.

### 2.6. Privacy-Aware Agent Boundaries

Physiological signals can be sensitive, especially when they can reveal health-related state. Differential privacy provides important background for formal privacy guarantees in data analysis and machine learning [70,71]. Federated learning provides another relevant privacy-oriented learning direction by reducing the need to centralize raw user data [72,73]. Privacy-attack studies also motivate careful data-minimization boundaries for learned systems [74,75]. Outside formal differential-privacy guarantees, prior work has also shown that raw motion-sensor data can be transformed on-device into a bounded representation that limits which inferences a downstream application can draw, without requiring a fully formal privacy model [76]. RideSafe-AI does not claim to implement differential privacy or federated learning in the current prototype. Instead, it places a practical privacy boundary between raw sensing and agent-level reasoning. The agent receives bounded summaries rather than raw streams, and explicit privacy guards in the Python reference (input and output) and the ESP32-S3 firmware (input) reject raw stream keys such as heart_rate_series, bvp_series,eda_series, acc_series, or direct identifiers.

### 2.7. Human-AI Decision Support and Trust Calibration

RideSafe-AI is designed as advisory decision support rather than automated control. This distinction matters because human–automation research shows that users may over-rely on automated aids, miss contradictory evidence, or lose situation awareness when automation is opaque or appears overly authoritative [14,15,17,19]. Human–AI interaction guidelines likewise emphasize communicating system capabilities, limitations, confidence, and uncertainty in ways that support appropriate reliance [77]. Abstaining from an uncertain prediction, rather than forcing a classifier to always output a label, is itself an established machine learning pattern with its own evaluation methodology [78]. For this reason, the proposed agent reports advisory states, confidence, uncertainty, motion-compromised abstention, and privacy-guard blocks instead of presenting a direct diagnosis or control command.

### 2.8. AI Reporting, Validation, and Evaluation Metrics

Because RideSafe-AI is an early-stage decision-support artefact, the paper separates reporting guidance from validation claims. Activity-recognition and model-evaluation literature motivates subject-wise validation, uncertainty reporting, and metrics beyond accuracy, especially under class imbalance, overlapping windows, and limited sample sizes [79,80,81,82]. These concerns are reflected in the held-out/LOSO reporting, confidence intervals, class-level metrics, ablations, and explicit claim-boundary tables used in the present manuscript.

Table 1 summarizes the architectural distinctions between RideSafe-AI and the closest prior studies discussed above.

## 3. RideSafe-AI Architecture

### 3.1. Design Principles and Evidence Separation

RideSafe-AI is a bounded, rule-governed decision pipeline built around one design principle: elevated physiology during riding is ambiguous until four separately-computed evidence sources—data quality, physical exertion/load, public-dataset stress-label evidence, and motion reliability—have each been evaluated on their own terms. Rather than a single classifier mapping sensor features directly to a rider-facing alert, each evidence source contributes a status, score, or confidence value, and a deterministic fusion step combines these into one of a fixed set of advisory states. RideSafe-AI is implemented as a hierarchical edge-agent architecture, independently re-implemented on three platforms: a Python reference implementation, an Android phone (Java), and an ESP32-S3 microcontroller (C++). This section describes the architecture’s components, data flow, interfaces, decision logic, and deployment tiers; dataset sources, training procedures, and validation protocols are described in Materials and Methods and Experimental Design below.

### 3.2. Bounded Input Representation

The agent operates on bounded feature windows rather than raw physiological streams. The current input schema contains ride features, stress features, and metadata. Ride features include mean heart rate, heart-rate drift, speed drop, cadence drop, and motion instability. Stress features include aggregate BVP, EDA, temperature, and acceleration statistics. Metadata includes the source and window duration. The Python reference rejects raw-stream fields and direct identifiers before model inference, and the ESP32-S3 applies an input-field guard. The Android tier constructs bounded inputs internally but does not implement an equivalent schema guard (Section 3.8).

### 3.3. Data Quality and Motion-Reliability Gating

The data-quality component checks whether a bounded feature window is suitable for agent reasoning. Missing required features and implausible bounded summaries, such as heart-rate values outside plausible physiological limits, cause a blocked decision rather than a rider-facing alert. Short windows are treated as usable with warnings.

The motion-reliability component evaluates whether device motion is high enough to reduce confidence in stress interpretation. This is a reliability-scoring step, not signal artefact correction: RideSafe-AI does not attempt to reconstruct or denoise a motion-corrupted signal; instead it reports a motion-reliability score and a physiology-confidence multiplier that downweights downstream evidence. If motion is high, the agent marks the window as motion_compromised and abstains from stress escalation until a cleaner window is available.

At the agent level, all three tiers use the same severity rule on bounded inputs: severity $=\max(m/0.8,s_{\mathrm{acc}}/0.09,r_{\mathrm{acc}}/1.5)$, where *m* is ride motion instability (g), and $s_{\mathrm{acc}}$ and $r_{\mathrm{acc}}$ are the standard deviation and range of acceleration magnitude (g). A window is motion_compromised (confidence multiplier 0.55) when any term reaches 1, watch_motion (multiplier 0.75) at m≥0.4, $s_{\mathrm{acc}}\geq 0.05$, or $r_{\mathrm{acc}}\geq 0.8$, and motion_ok (multiplier 1.0) otherwise. The tiers differ in their motion inputs. The ESP32-S3 first summarizes 2 s MPU6500 (TDK InvenSense, San Jose, CA, USA) windows sampled at 50 Hz and marks a frame window as compromised when dynamic RMS ≥0.18 g, jerk RMS ≥6 g/s, or range ≥1.5 g; these frame summaries are then passed to the agent rule. The Android tier uses the ESP32-S3 summaries when they are fresh and otherwise derives a motion proxy from the watch accelerometer and gyroscope. The Python reference receives motion summaries in its input schema. The tiers therefore share the rule but not the input signals, and they are not numerically equivalent.

### 3.4. Exertion and Stress Evidence Layers

The exertion/load proxy tool uses a weighted *k*-nearest-neighbour model trained on PAMAP2-derived activity/load proxy windows. The safe interpretation is physical load or exertion proxy estimation, not fatigue diagnosis and not psychological stress recognition.

The stress-label tool uses a weighted *k*-nearest-neighbour model trained on WESAD low/high stress feature windows. The output is a public-dataset stress-label classification and is not a clinical diagnosis or validated rider-stress detector.

The two weighted-kNN models run only in the Python reference implementation. Their branch confidence is the inverse-distance-weighted vote share of the predicted class among the *k* nearest neighbours. The Android and ESP32-S3 tiers do not execute kNN, RF, SVM, or XGBoost. They use a fixed rule-based scorer: a weighted sum of clipped heart-rate, heart-rate-drift, speed-drop, and cadence-drop terms for exertion/load, and of clipped BVP and EDA terms for the stress label, with fixed mappings from score to label confidence. The smartwatch used in the technical rides does not supply BVP, EDA, or skin-temperature summaries to the phone. In live Android operation, these stress-feature inputs are therefore generated from a single heart-rate-based stress index, and the speed- and cadence-drop inputs are generated from heart-rate drift. The live stress-label branch during riding thus reflects a heart-rate heuristic, not the WESAD-trained model.

### 3.5. Conservative Fusion, Uncertainty, and Abstention

The fusion layer combines data quality, exertion/load proxy evidence, stress-label evidence, and motion reliability through a deterministic rule sequence rather than a learned model. Three mechanisms make fusion conservative: high-risk model labels below an alert-confidence threshold are downgraded to a conservative watch state; stress-label evidence that coincides with motion-compromised input is treated as unreliable regardless of its own reported confidence; and a global fused-confidence floor sets an abstention flag and high uncertainty independent of which individual branch is uncertain.

Table 2 lists the three decision thresholds. Source inspection confirmed the same values in the Python reference, Android Java, and ESP32-S3 C++ implementations. These values are heuristic prototype parameters; they were not optimized using independently labelled riding data. The implementations also contain further heuristic constants, including data-quality confidences (0.92 good, 0.68 usable with warnings, 0.25 blocked), the motion multipliers above, a plausible heart-rate range of 35–220 bpm, and, on Android and ESP32-S3, a stress-index threshold of 0.70 for the device-level state.

When exertion and stress evidence disagree, the following precedence applies. If both branches report a high-risk label and both reach the alert-confidence threshold, the rider state is combined_high_load; if only the stress branch reaches the threshold, the rider state is possible_stress; if only the exertion branch reaches the threshold, the rider state is high_physical_load; if both branches report a high-risk label but neither reaches the threshold, the rider state is uncertain_load_watch. These rules are those of the Python reference; one precedence difference in the Android and ESP32-S3 implementations is described in Section 3.8. Table 3 gives the complete mapping from this intermediate rider-state assessment to the final safety-status output.

The low, moderate, and high uncertainty categories are deterministic decision-status indicators derived from the fusion and reliability rules. They do not quantify a probability of error or estimate epistemic or aleatoric uncertainty. Fused confidence is a continuous rule-derived score, $c_{\mathrm{fused}}=\min\left(c_{\mathrm{quality}},c_{\mathrm{exertion}},c_{\mathrm{stress}}\right)\times m_{\mathrm{motion}}$, where the three terms are the data-quality and branch confidences and $m_{\mathrm{motion}}$ is the motion multiplier. Uncertainty is categorical and is assigned by the first matching rule: high for blocked data quality, motion-compromised high stress-label evidence, or $c_{\mathrm{fused}}<0.45$; moderate for data-quality warnings, moderate motion, a branch confidence below 0.60, or $c_{\mathrm{fused}}<0.65$; and low otherwise. Abstention is a Boolean flag set with high uncertainty and records a machine-readable reason. The conservative safety status itself is produced by two separate rules, blocked data quality and motion-compromised high stress-label evidence (Table 3). A fused confidence below 0.45 sets the flag but does not change the safety status; this was confirmed in the software sensitivity checks reported in Section 6.10.

### 3.6. Safety Decision and Advisory Output

The final safety output belongs to a restricted state set:

     normal, watch, high_exertion, possible_stress,
      critical_watch, motion_compromised_watch,
         blocked, blocked_by_guard.

The output is intentionally conservative and non-clinical, and is advisory only: no component of the architecture issues e-bike or e-scooter control commands.

### 3.7. Privacy and Data-Minimization Boundary

RideSafe-AI enforces a practical data-minimization boundary, not a formal privacy guarantee. Before the boundary, sensing may include raw heart-rate/PPG samples, accelerometer streams, GPS coordinates, and device or participant identifiers. Across the boundary, only bounded summaries, categorical evidence, reliability scores, and reason codes are permitted. Rejected fields are identified by exact key name and by pattern (for example, keys ending in _series, _stream, _values, or beginning with raw_), checked at both the input and output stage in the Python reference. This mechanism is confirmed by inspection in the Python reference implementation and independently in the ESP32-S3 firmware, which implements its own privacy-guard check and returns blocked_by_guard when a forbidden field is present. No privacy-guard check was found in the Android source code. The Android tier builds its agent input internally from watch and ESP32-S3 summaries, but its inputs, outputs, and logs are not screened by an equivalent guard.

### 3.8. Cross-Tier Implementation: Python, Android, and ESP32-S3

**Figure 1:**
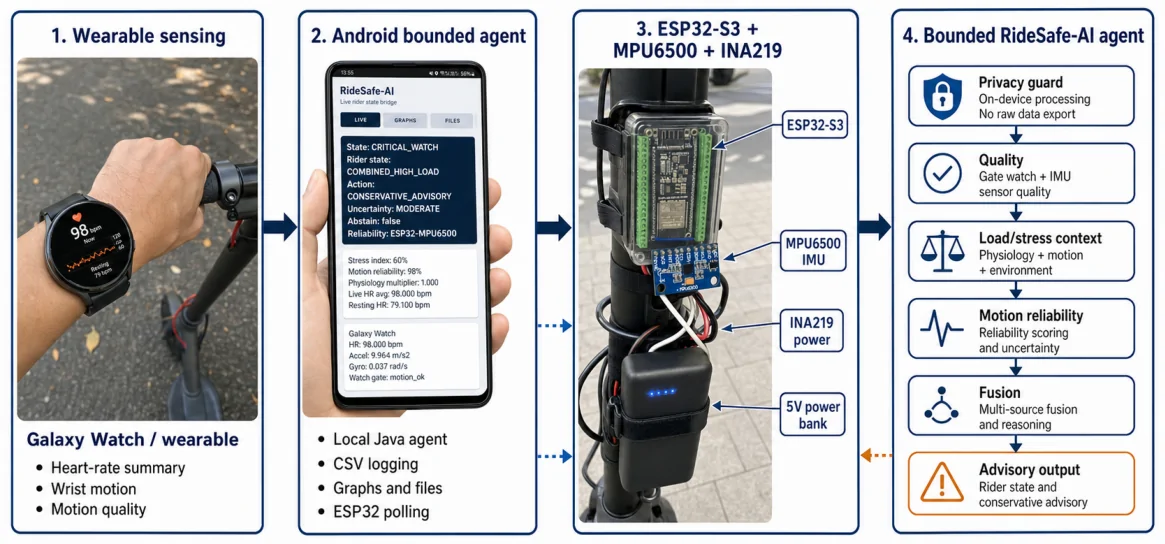
Hierarchical edge-agent architecture used in the current RideSafe-AI prototype. The Android phone runs a Java agent and comparison display, while the ESP32-S3 node runs a constrained C++ agent for motion reliability and local advisory-state generation from aggregate inputs. The Python implementation remains the reproducible reference and reporting layer. The rightmost panel is a conceptual summary of the decision logic shared across tiers, not a fourth deployment tier and not a claim of identical implementation across Python, Android, and ESP32-S3.

Figure 1 summarizes the current deployment split.

The Python reference implementation used to produce the results reported in this manuscript executes a fixed sequence of deterministic function calls over classical machine learning classifier outputs; it does not invoke a language model. The ESP32-S3 component is likewise not presented as a Python or LLM-style agent: it is a bounded embedded C++ agent that performs efficient motion-window extraction, privacy/data-quality checking, lightweight scoring, motion reliability, fusion, uncertainty/abstention, and advisory-state output from aggregate features. The Android phone app also implements the agent in Java for live wearable-driven decision support and side-by-side comparison with the ESP32 endpoint, while the Python implementation remains useful for reproducible reference evaluation, longer reports, and comparison logging.

**Figure 2:**
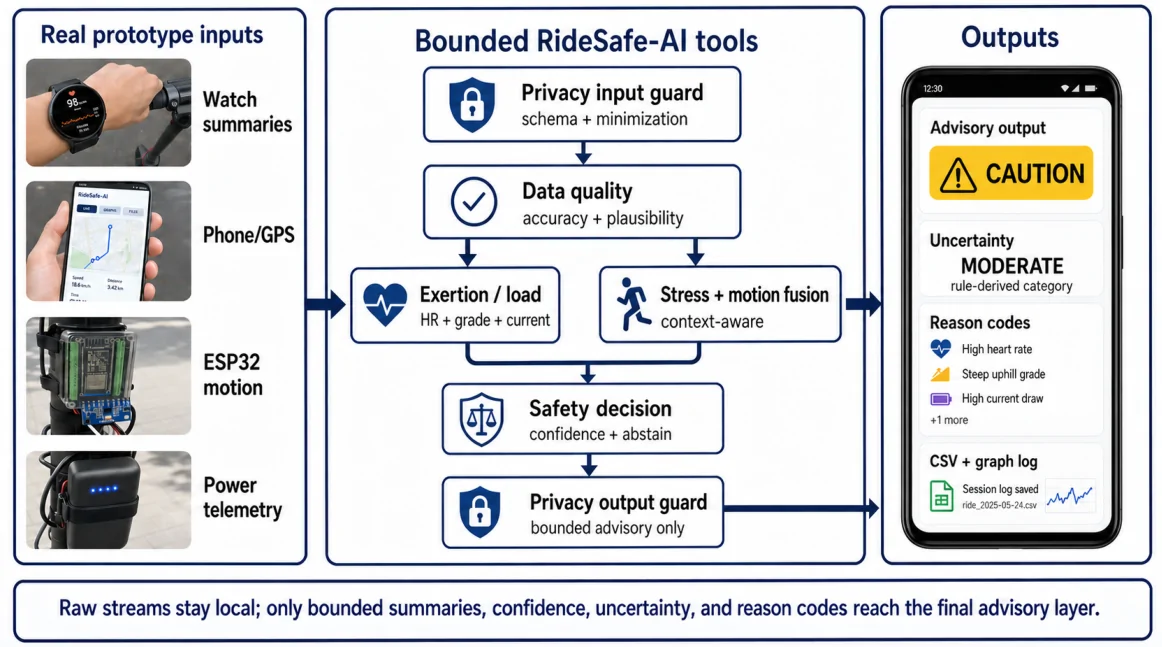
Conceptual workflow of the Python reference path. The displayed uncertainty is a rule-derived categorical status, not a calibrated probability or percentage. The input/output guard boxes represent the Python reference; the ESP32-S3 implements an input guard, whereas Android constructs bounded inputs internally without an equivalent guard (Table 4). The GPS-terrain and power-telemetry reason codes shown in the example output (e.g., steep uphill grade, high current draw) illustrate a planned output format; their active use as decision inputs is not confirmed in the current implementation and is scoped as logging readiness only (see Experimental Design and Discussion).

Figure 2 shows the agent-level workflow. The key design choice is that raw physiological streams are not passed to the final decision layer. Each tool contributes a status, score, confidence, or reason code, and the final safety output is generated from these bounded values.

Across tiers, “shared architecture” refers to common processing stages and a common categorical output vocabulary. The uncertainty rules, the mapping to safety status, and the three thresholds in Table 2 match by source inspection. The rider-state fusion differs in one precedence rule: when the exertion label is medium and the stress label is high with confidence of at least 0.60, the Python reference returns possible_stress, whereas the Android and ESP32-S3 implementations return moderate_physical_load (safety status watch). The branch scorers (kNN in Python; a rule-based scorer on Android and ESP32-S3), the motion input signals, and the privacy guard (absent on Android) differ. Table 4 summarizes these differences. A matched-input test was performed on a host computer for this revision. The Android and ESP32-S3 decision functions were extracted verbatim from the archived source files and compiled on the host (Java 17 and g++ 13), with hardware and communication code replaced by minimal stubs. They were then run with the Python reference on identical inputs. First, for 4374 combinations of data-quality status, branch labels and confidences (0.40–0.95, including values at the 0.45, 0.60, and 0.65 boundaries), and motion levels, the Android and ESP32-S3 outputs were identical. Python agreed with both in uncertainty category and abstention for all combinations. Its rider state differed in 486 combinations and its safety status in 180, all caused by the precedence difference described above. Fused confidence differed by at most 0.001 in 258 combinations because of floating-point rounding. Second, on the 488 non-attack windows of the designed scenarios and the deterministic suite, each implementation running its own branch scorer produced the same safety status, rider state, and motion gate. Fused confidence agreed in only 29 windows and the uncertainty category in 415, reflecting the different branch scorers. These designed inputs lie away from decision boundaries, so this agreement does not generalize to riding data. Third, the Python and ESP32-S3 privacy guards gave the same decision for all 68 tested field names. Both accepted several sensitive-looking names that do not match their patterns, for example heartRateSeries, ppg_raw, latitude, and phone_number. This test verifies the decision code on identical inputs. It does not test the phone or microcontroller hardware, timing, or sensor inputs, so numerical and behavioral equivalence on the devices has not been established. In one approximately 10 min engineering-validation session, all 528 bounded feature windows with usable phone and ESP32-S3 outputs had matching safety-state, rider-state, and motion-gate categories. This observation is limited to that session and its input range. Because the phone and the ESP32-S3 apply the same rule-based scorer and fusion rules to aggregate inputs sent by the phone, this agreement mainly shows consistent execution of shared logic rather than independent confirmation. It does not establish agreement on intermediate scores, boundary cases, other riding conditions, or other participants.

**Figure 3:**
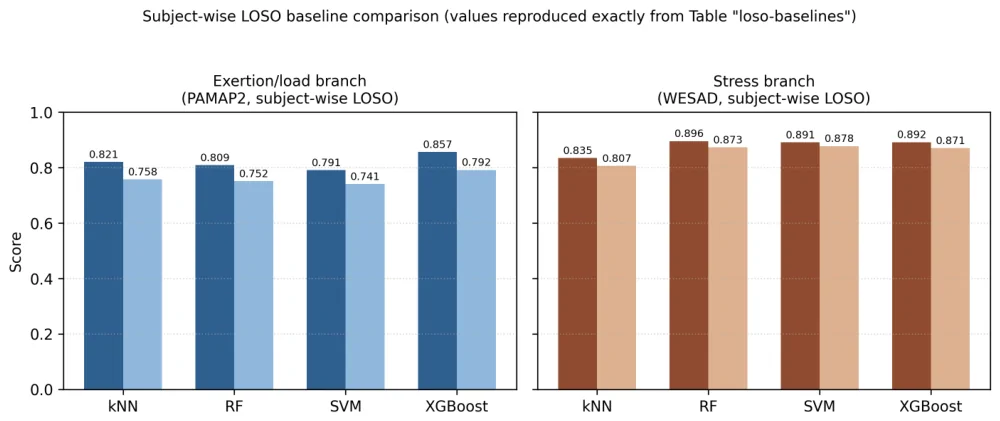
Subject-wise LOSO pooled accuracy and fold-level macro-F1 for the exertion/load branch (PAMAP2) and the stress branch (WESAD),reproduced exactly from the LOSO baseline comparison in Section 6. This figure supports the architecture-feasibility and public-dataset branch-modelling claims only; it is not evidence of real-world rider stress detection or safety impact. Dark bars show pooled accuracy and light bars mean fold-level macro-F1; blue denotes the exertion/load branch (PAMAP2) and brown the stress branch (WESAD).

Figure 3 summarizes the subject-wise LOSO accuracy and macro-F1 evidence for the exertion and stress branches,reproduced exactly from the LOSO baseline comparison in Section 6.1.

### 3.9. Architecture Claim Boundary

RideSafe-AI is positioned as non-clinical rider-facing decision support for research prototyping, following DECIDE-AI-inspired principles for intended use, deployment context, human-facing output, safety boundaries, and limitations [27]. It accepts bounded feature windows only and returns a restricted advisory-state set, leaving interpretation and action to the rider or researcher. Raw-stream and identifier rejection is enforced by the Python reference and, for incoming fields, by the ESP32-S3; Android constructs bounded inputs internally without an equivalent guard. It is not intended to diagnose stress, fatigue, illness, impairment, or emergency state; it is not a medical device; and it is not an autonomous safety controller. The current evidence supports architecture feasibility and cross-tier implementation feasibility; it does not establish field-calibrated motion-gate performance, validated real-world rider-stress detection, or safety-effectiveness (see Limitations).

## 4. Materials and Methods

The methodology follows a design science research approach because the primary contribution is the design, implementation, and pre-deployment evaluation of a technological artefact. The artefact is the RideSafe-AI architecture, which integrates bounded physiological feature summaries, exertion/load proxy estimation, public-dataset stress-label classification, motion-reliability gating, privacy guards, and conservative advisory-state fusion; its components, data flow, and decision logic are described in Section 3 above. The process is operationalized through problem identification, design-objective definition, artefact development, prototype demonstration, multi-part evaluation, and communication of claim boundaries. The prototype hardware comprised a Samsung Galaxy Watch7 smartwatch and a Samsung Galaxy A23 smartphone (Samsung Electronics, Suwon, Republic of Korea), an ESP32-S3 microcontroller, an MPU6500 inertial measurement unit, and an INA219 current and power monitor. The Python reference used Python 3.13.1 (Python Software Foundation, Wilmington, DE, USA) with scikit-learn 1.9.0 and XGBoost 3.3.0; the Android applications were written in Java and built with Gradle 8.9; and the ESP32-S3 firmware was written in C++ using the Arduino-ESP32 core 2.0.14.

## 5. Experimental Design

The evaluation is designed as a pre-deployment assessment. It includes public-dataset model evaluation, subject-wise and leave-one-subject-out (LOSO) validation, classical baseline comparison, designed bounded rider-state scenarios, ablation studies, threshold sensitivity analysis, robustness/privacy tests, repeated runtime benchmarking, ride-context logging metrics, and live edge-device feasibility checks.

### 5.1. Public Dataset Model Evidence

PAMAP2 is used for exertion/load proxy modelling. WESAD is used for low/high public-dataset stress-label classification. The two branches are kept separate to avoid claiming that physical activity data alone provides psychological stress labels. PAMAP2 features are extracted as 10 s non-overlapping windows from nine subjects, built directly from each source file’s native per-sample timestamps without resampling, decimation, or interpolation, so window contents reflect the dataset’s own native sampling structure. The three-level exertion/load proxy label is the higher of two classes: an intensity class assigned to each PAMAP2 activity (low, medium, or high) and a heart-rate class (medium at a window mean of at least 105 bpm, high at 135 bpm or more). Because mean heart rate is also a model input, part of the label depends on an input; the heart-rate class raised the label above the activity class in 60 of 1969 windows (3.0%). PAMAP2 contains no bike speed or cadence, so the speed-drop and cadence-drop inputs are set to zero for all PAMAP2 windows. WESAD wrist features are extracted as 60 s windows with a 30 s step from 15 subjects, retaining windows only when at least 80% of the label samples agree with the dominant baseline or stress condition. Public-data windows are evaluated with the existing held-out model split and with LOSO validation using the subject identifier in each feature table. In LOSO evaluation, all windows from the held-out subject are excluded from training, and model fitting and feature scaling are repeated using only the training subjects. This is especially important for WESAD because overlapping windows from the same subject must not appear across train and test folds; subject-wise validation is recommended in wearable activity-recognition settings because random window splits can overestimate generalization when samples from the same person are correlated [49]. The reported confidence intervals use Wilson intervals for pooled held-out or pooled LOSO accuracy and fold-level 95% confidence intervals for LOSO mean accuracy and macro-F1. Macro-F1 is the unweighted mean of the per-class F1 scores, balanced accuracy is the unweighted mean of the per-class recalls, and the exercise false-stress rate (FSER) is the fraction of exercise windows predicted as stress. The LOSO baseline comparison uses weighted kNN, random forest (RF), radial-basis SVM, and XGBoost classifiers with the same feature windows and subject folds. The weighted-kNN models use k=15 for PAMAP2 and k=11 for WESAD in both the held-out and LOSO evaluations, with no per-fold re-tuning of *k*; neighbour votes are weighted by inverse distance (1/d). The comparator RF, SVM, and XGBoost models use fixed hyperparameters (RF: 300 trees, unlimited depth; SVM: radial-basis kernel, C=1.0, scale-based gamma; XGBoost: 150 estimators, maximum depth 3, learning rate 0.05) and a fixed random seed (42) for any stochastic component; none of these hyperparameters are re-tuned per LOSO fold. RF and SVM use balanced class weights; kNN and SVM include feature standardization fitted on the training fold. The PhysioNet experiments use separately fixed settings: distance-weighted kNN with k=7; RF with 80 trees, maximum depth 14, minimum leaf size 2, and balanced-subsample class weights; linear SVM with C=1.0 and balanced class weights; and XGBoost with 80 estimators, maximum depth 3, and learning rate 0.05.

The public datasets provide evidence for specific model components. PAMAP2 supports activity-derived exertion/load proxy modelling, whereas WESAD supports laboratory stress-label classification. Neither dataset establishes accuracy during e-bike or e-scooter riding. Differences in activities, sensor placement, vibration, environmental conditions, and label definitions may affect transfer. The complete system therefore requires evaluation on representative riding data with suitable independent reference measurements.

An external-data extension and audit layer has now been implemented in the reproducibility code before recruited rider collection. The layer includes a machine-readable support-dataset registry, a fixed RideSafe-AI bounded collection schema, a schema validator, a protocol-aware PhysioNet stress–exercise adapter, a flattened CSV adapter for GiroE-style e-bike smartwatch data, and an implementation-evidence report generator. The older local-derived PhysioNet support CSV contains 3425 schema-valid windows, but all of those windows are labelled as stress with unknown exertion labels; it is therefore retained only as a negative label-sufficiency audit. The official PhysioNet v1.0.1 package was then acquired and reprocessed from STRESS, AEROBIC, and ANAEROBIC folders with tags.csv event markers, stress-level files, and subject metadata; windows are 10 s in length with a 5 s step (50% overlap), applied uniformly across all reprocessed protocols and the FSER comparison. This produced 36,472 bounded windows from 36 subjects, including stress, rest/non-task, aerobic, and anaerobic labels for LOSO checks on this independent public-dataset source; Section 6.3 audits exactly which of the 36 subjects contribute which classes, because not every subject contributes both sides of every binary comparison. The GiroE adapter has generated 3336 schema-valid e-bike support windows from GNSS/speed/heart-rate/temperature records for route-context and motion-feature alignment, again without stress-label validation; participant identity is not preserved in this conversion (the subject identifier is recorded as unknown for all 3336 windows), so a GiroE participant count is not reported. The cycling-stress and e-scooter/bicycle-lane sources are represented in the registry as protocol-support evidence, because directly reusable raw training data were not included in the local reproducibility package. Because these external sources differ in modality and label type, the implemented layer is used for transfer, schema validation, and protocol design rather than as a single pooled ground-truth benchmark.

### 5.2. TRIPOD + AI-Oriented Model Reporting

The model-level reporting follows TRIPOD+AI-style transparency for data sources, predictors, labels, validation metrics, and intended-use boundaries [28]. PAMAP2 is the physical exertion/load branch, WESAD is the public stress-label branch, official PhysioNet is a protocol-reconstructed stress/exercise feature-set check, and GiroE/cycling/e-scooter sources are context or protocol support only. These branches are not pooled into a single rider-stress ground truth.

### 5.3. Terminology: Feature-Set Comparison Versus Agent Ablation

The manuscript uses two evaluations that sound similar but test different things, and they are kept terminologically separate throughout.

The *PhysioNet feature-set experiment* is a same-observation classical-model comparison on the official PhysioNet v1.0.1 stress/exercise windows: for each held-out subject and each of four models (kNN, RF, linear SVM, XGBoost), the same LOSO fold is scored twice, once using only stress-sensor features (BVP, EDA, temperature) and once using the full feature set (stress sensors plus HR and accelerometer summaries). It quantifies how much additional exertion-related signal (HR level/drift and motion-derived accelerometer summaries) reduces the exercise false-stress rate within a single classical-model pipeline. It is public laboratory evidence for exertion-aware discrimination; it does not run the RideSafe-AI agent, its motion-reliability gate, its fusion logic, or its privacy guard.

The *RideSafe-AI agent ablation* (Section 6.8) is a component-removal test of the full agent on designed rider-state scenarios, comparing stress-only, stress plus exertion/load proxy, stress plus exertion/load plus motion gate, and the full agent with privacy guard. It measures agent-level safety-status agreement, not classifier accuracy, and it does not use PhysioNet data.

These two evaluations are complementary, not interchangeable. The PhysioNet feature-set experiment strengthens the empirical case that exertion-related physiological and motion information reduces false stress classification under subject-independent evaluation. It does not reproduce the complete bike-side RideSafe-AI pipeline, because the official PhysioNet dataset contains no MPU6500 bike-frame motion, GPS terrain, or INA219 power context, and it does not exercise the agent’s motion-reliability gate, fusion, uncertainty/abstention, or privacy-guard logic. The RideSafe-AI agent ablation is designed-scenario evidence for the decision logic itself, not an external subject-independent dataset result. RQ2 (Section 1.2) and the research-question summary (Section 6.14) are answered using both evaluations together, with this boundary stated explicitly rather than implied.

### 5.4. Designed Scenario Evaluation

Nine bounded scenarios are used to test agent-level decision logic: normal low load, moderate physical load, high physical load, possible stress with clean motion, combined high load, motion-compromised stress, short window warning, out-of-range heart-rate summary, and privacy-guard rejection. These are not real-world rider validation trials. They are controlled bounded input scenarios used to test safety logic and failure handling.

The expected scenario outcomes are rule-derived before execution from the stated design requirements: physiological elevation should not automatically become a stress alert when exertion is high; high motion should reduce confidence in stress-label escalation; short or implausible inputs should warn or block rather than over-alert; and raw-stream or identifier leakage should be rejected by the privacy guard. The scenario labels are therefore treated as a designed benchmark for traceability and failure handling, not as external ground truth for field performance.

### 5.5. Deterministic Scenario Verification

The nine scenario templates are expanded deterministically into 500 bounded feature windows. Expected outputs are derived from the decision specification. The verification suite includes normal, exertion/load, possible stress-label, combined-load, motion-compromised, short-window, implausible-heart-rate, and privacy-attack cases. It checks conformance to the specified rules for the generated inputs. The 500 windows do not represent 500 independent rider observations, and the suite does not establish exhaustive coverage of operating conditions or decision boundaries.

### 5.6. Ablation Study

Four variants are compared: stress-only, stress plus exertion/load proxy evidence, stress plus exertion/load plus motion gate, and full RideSafe-AI with privacy guard.

The stress-only variant is intentionally a weak decision-logic lower bound rather than a proposed deployment model. Its role is to show what errors remain when exertion/load evidence, motion confidence control, and privacy guarding are removed. Threshold sensitivity is evaluated separately so that the ablation does not conflate component removal with one particular alert threshold.

### 5.7. Robustness and Privacy Tests

The robustness suite checks missing heart-rate summaries, missing stress features, raw stream leakage attempts, high-motion stress, short windows, and implausible heart-rate values.

### 5.8. Threshold Sensitivity

The original analysis swept only the alert-confidence threshold, from 0.40 to 0.80, on the nine designed scenarios. For this revision, a software sensitivity check varied each of the three parameters separately in the Python reference agent while holding the other two at their defaults: the alert-confidence threshold (0.40–0.80), the abstention floor (0.30–0.60), and the moderate-uncertainty boundary (0.55–0.80). Each setting was run on the nine designed scenarios and on the 500-window deterministic suite. The check records agreement with the rule-derived expected safety status, the safety-status distribution, the abstention flag, and the uncertainty categories. It uses a copy of the agent in which the two literal constants were replaced by parameters, without changing the original source; outputs of this copy at the default values were identical to those of the original agent. Because the inputs and expected outputs are rule-derived, this is a software sensitivity check. It is not threshold optimization or field validation; selecting empirical operating points requires independently labelled riding data.

### 5.9. Runtime and Edge Feasibility

Runtime is measured using repeated execution of the Python reference agent and live-device feasibility checks. The edge-feasibility checks compile and upload the ESP32 firmware, verify Android phone/watch builds, and confirm that the Galaxy Watch stream reaches the Android bridge while the phone is connected to the ESP32 access point. The ESP32-S3/MPU6500 firmware emits motion-reliability windows and serves status, command, and bounded-agent HTTP endpoints. The Android phone app includes a Java agent and an ESP32-agent comparison panel; a live normal-state check showed matching phone and ESP32 agent states. A fixed-duration phone-side logging run then measured repeated ESP32 /status and /agent endpoint calls over an approximately 10 min session. INA219 telemetry provides direct short-window ESP32 power observations in the bench check and technical ride log, while longer-duration field energy profiling still requires additional connected live-device measurement.

### 5.10. Ride-Context Logging and Self-Labelling Protocol

The latest Android implementation adds a ride-context CSV schema for technical validation of the full prototype. Each auto-saved row contains smartwatch physiology and wrist motion, phone-agent state, ESP32-agent state, ESP32/MPU6500 motion reliability, INA219 voltage/current/power telemetry, GPS latitude/longitude, altitude, speed, provider status, road-grade estimate, terrain segment, and an auto_segment field. The auto_segment field is a first-pass self-label generated from GPS speed, altitude/grade, watch motion, ESP32 motion reliability, jerk, and power/motion context. It can take values such as stationary, smooth_road, rough_road_or_mount_issue, uphill, downhill, hard_accel_or_brake, location_waiting, or unknown.

This self-labelling layer is used only as a technical segmentation aid, not as ground truth. Its purpose is to reduce manual annotation burden and to make future rider-context validation measurable. The current single-participant technical log demonstrates automatic segment generation and quality reporting, while controlled validation should still separate repeated route conditions such as stationary table/deck placement, smooth flat riding, rough surface riding, uphill riding, downhill riding, and controlled hard acceleration/braking. For each segment, the analysis script reports GPS fill rate, auto-segment counts, agent agreement/disagreement, possible false stress escalations, energy-safety flags, and mount-quality flags.

Three descriptive implementation diagnostics connect ride logging to the later validation protocol. First, the logging analysis flags windows in which stress-label outputs coincide with uphill, rough-road, hard-braking, high-current, or high-power context. These are heuristic context candidates, not identified stress misclassifications, because the technical rides contain no independent stress ground truth. Second, the analysis reports whether elevated physiology coincides with road-load indicators, frame vibration, or lower-load smooth riding. Third, repeated ESP32 motion_compromised states during stationary or smooth-road auto segments are used to diagnose sensor-mount reliability. These diagnostics define checks for future ethics-approved rider studies; they do not estimate rider-stress accuracy or safety impact.

## 6. Results

### 6.1. Model-Level Results

The Python reference implementation uses dependency-light weighted-kNN models because their JSON representation can be inspected and executed without a full scientific Python stack. This is an implementation-convenience choice, not evidence that kNN provides the best predictive or resource trade-off. RF, SVM, and XGBoost are included as comparative public-dataset baselines. As an exploratory paired fold-level comparison against kNN, XGBoost improved PAMAP2 fold accuracy by 0.032 (95% CI 0.005–0.059), while RF and SVM did not improve PAMAP2. For WESAD, SVM improved fold accuracy by 0.057 (95% CI 0.015–0.099); RF and XGBoost also had positive mean deltas (0.061 and 0.057), but their 95% CIs included zero. These public-dataset comparisons do not establish superiority in a riding context.

To examine the resource side of this trade-off, the four classifiers were profiled under identical host conditions (Table 5). Each model used the same features, LOSO folds, and hyperparameters as the LOSO baseline comparison in Section 6.1. For every fold, the fitted estimator was serialized to measure stored size, and every held-out window was then predicted individually to measure single-window latency, including the scaling step of the kNN and SVM pipelines. Each dataset–model pair ran in a separate process, and peak memory is reported as the increase in process working set over the post-data-loading baseline. Feature extraction is shared by all models and was not re-timed. The host was a laptop with an Intel Core i5-1245U processor and 15.6 GB RAM (Windows 11, Python 3.13.1, scikit-learn 1.9.0, XGBoost 3.3.0). Latency statistics are pooled over one pass through all held-out windows (1969 PAMAP2 and 883 WESAD windows).

On this host, kNN did not have the lowest latency or stored size. SVM was the smallest and fastest model on both datasets. XGBoost combined the highest PAMAP2 accuracy with a moderate size and sub-millisecond median latency. RF was the largest and slowest configuration because its 300 unpruned trees were evaluated through the general-purpose library interface. The dependency-light JSON weighted-kNN used by the Python reference agent occupied 223 kB (PAMAP2) and 256 kB (WESAD) and had median/p95 latencies of 2.49/4.40 ms and 2.72/5.26 ms over 1000 calls with a fixed input window. These results do not support kNN as the most resource-efficient model. They also do not transfer directly to microcontrollers, where model size, integer or floating-point support, and code generation dominate. Energy per inference was not measured. On-device comparison of the four classifiers would require porting each model to the ESP32-S3 or Android tier and instrumenting its energy use; this was not performed.

Table 6, Table 7, Table 8 and Table 9 report the model evidence in four layers: held-out branch performance, class-level held-out behavior, pooled LOSO confusion matrices, and subject-wise LOSO baseline comparisons. The purpose is to show both headline accuracy and the main error patterns before interpreting the branch models inside the agent.

The headline split intentionally differs across branches: PAMAP2 provides only 9 subjects, so a subject-wise held-out split at this stage would leave a very small, high-variance test partition, whereas WESAD’s 15 subjects support a stable subject-wise held-out estimate as the headline number. This is a reporting-convenience choice for Table 6, not a claim that PAMAP2 was evaluated only with a random window-level split: Table 9 reports full leave-one-subject-out validation for both PAMAP2 and WESAD across all four model families, and that subject-independent comparison, not the random-split headline number, is the evidence used to support the exertion/load branch.

Overall, the model tables support the use of PAMAP2 as an exertion/load proxy branch and WESAD as a public stress-label branch, while preserving the key boundary that neither table validates real-world rider stress detection. In particular, the WESAD accuracy of 0.850 in Table 6 describes classification of low/high laboratory condition labels under the reported subject-wise held-out split. It does not measure the complete RideSafe-AI system or establish accuracy for detecting psychological stress during riding. The class metrics and LOSO matrices also motivate the later fusion design: the stress branch is informative, but it should be moderated by exertion, motion reliability, and data-quality checks.

### 6.2. External Evidence-Gating Audit

The reproducibility-audit script was added to prevent external-dataset overclaiming. It recomputes local artefacts, checks label availability, and writes status files to the reproducibility package. A later protocol-aware PhysioNet validation script preserves the negative audit of the older 3425-window all-stress support file but also reprocesses official PhysioNet v1.0.1 STRESS, AEROBIC, and ANAEROBIC folders with tags.csv event markers, stress-level files, and subject metadata. The completed status items most relevant to the paper are as follows: 36,472 PhysioNet protocol-labelled windows from 36 subjects, subject-held-out stress/exercise modelling, a subject-structure audit, participant-level FSER statistics, GiroE context-window preparation, direct privacy-guard tests, WESAD bounded-feature attribute inference, and an expanded HR reconstruction benchmark. Long-duration Android–ESP32 reconnect and field-energy testing remain ready-to-run but not completed. The PhysioNet results are public laboratory protocol-window evidence for stress/exercise separation; they are not real e-bike or e-scooter rider-stress validation. Table 10 summarizes these PhysioNet results.

### 6.3. PhysioNet Subject-Structure Audit

Before reporting P1 as a “36-subject” result, a subject-structure audit script was added to verify exactly which participants contribute which classes, because P1 pools stress windows from some subjects and exercise windows from others rather than always both. Table 11 reports the resulting counts.

The local reconstruction excludes subjects S02 and f07 from the STRESS protocol only, because the official PhysioNet notebook documents duplicate or invalid sensor streams for those two stress sessions; this is why the local stress-participant count (34) is below the published count (36), while the aerobic (30) and anaerobic (31) participant counts match the participant counts reported in the Scientific Data descriptor exactly [25], which is consistent with the local protocol reconstruction.

**Figure 4:**
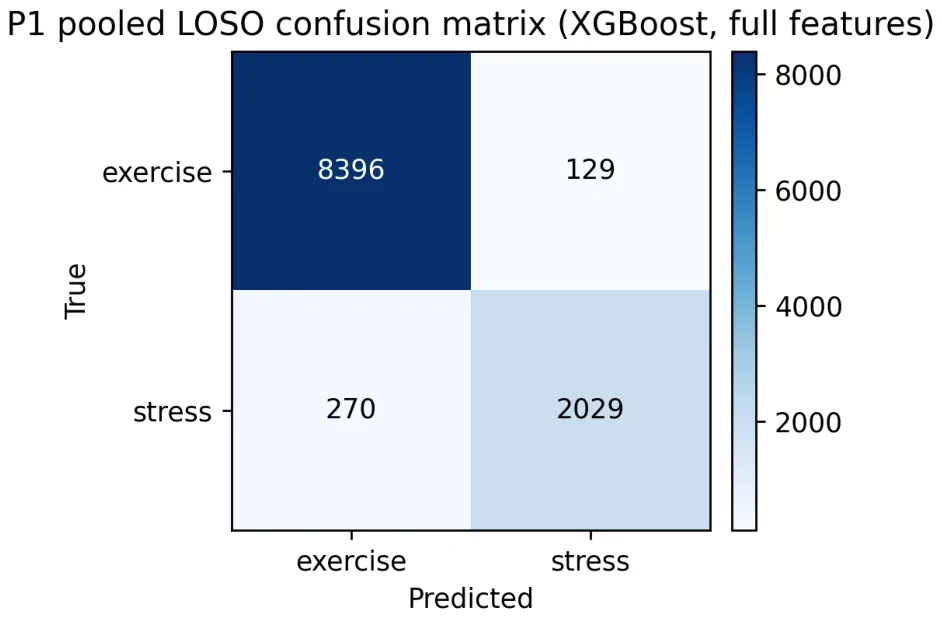
Pooled LOSO confusion matrix for P1 stress-versus-exercise (XGBoost, full features). The underlying CSV is provided in the reproducibility package.

Critically, not every P1 held-out fold contains both classes. Subjects S02 and f07 contribute exercise windows but no stress windows (excluded above); subjects f14–f18 contribute stress windows but no aerobic/anaerobic session was recorded for them. Their LOSO held-out fold is therefore single-class: for S02 and f07, every held-out prediction has true label “exercise”, and for f14–f18, every held-out prediction has true label “stress”. P1 accuracy, balanced accuracy, macro-F1, AUROC, and AUPRC are computed from *pooled* out-of-fold predictions across all 36 held-out folds for each (feature set, model) pair, not by averaging per-fold metrics, so these headline numbers remain well defined even though seven individual folds cannot by themselves exercise both directions of the binary decision. Per-fold metrics are still written to the reproducibility package for transparency, and per-fold macro-F1/AUROC for those seven folds is degenerate by construction (a single-class fold cannot have a meaningful two-class macro-F1); Figure 4 and the per-participant macro-F1 plot in the reproducibility package show this directly, with the seven single-class subjects visibly lower (0.34–0.50) than the 29 dual-condition subjects (0.75–1.00) purely as an artefact of having only one true class to score against.

As a sensitivity check, P1 was re-fit restricted to only the 29 dual-condition subjects, excluding the other 7 from both training and evaluation (not just filtering existing predictions). Table 12 compares the two runs for the full feature set; results are closely similar, so the reported P1 headline numbers do not appear to be an artefact of the seven single-class folds.

### 6.4. FSER Statistical Analysis

**Figure 5:**
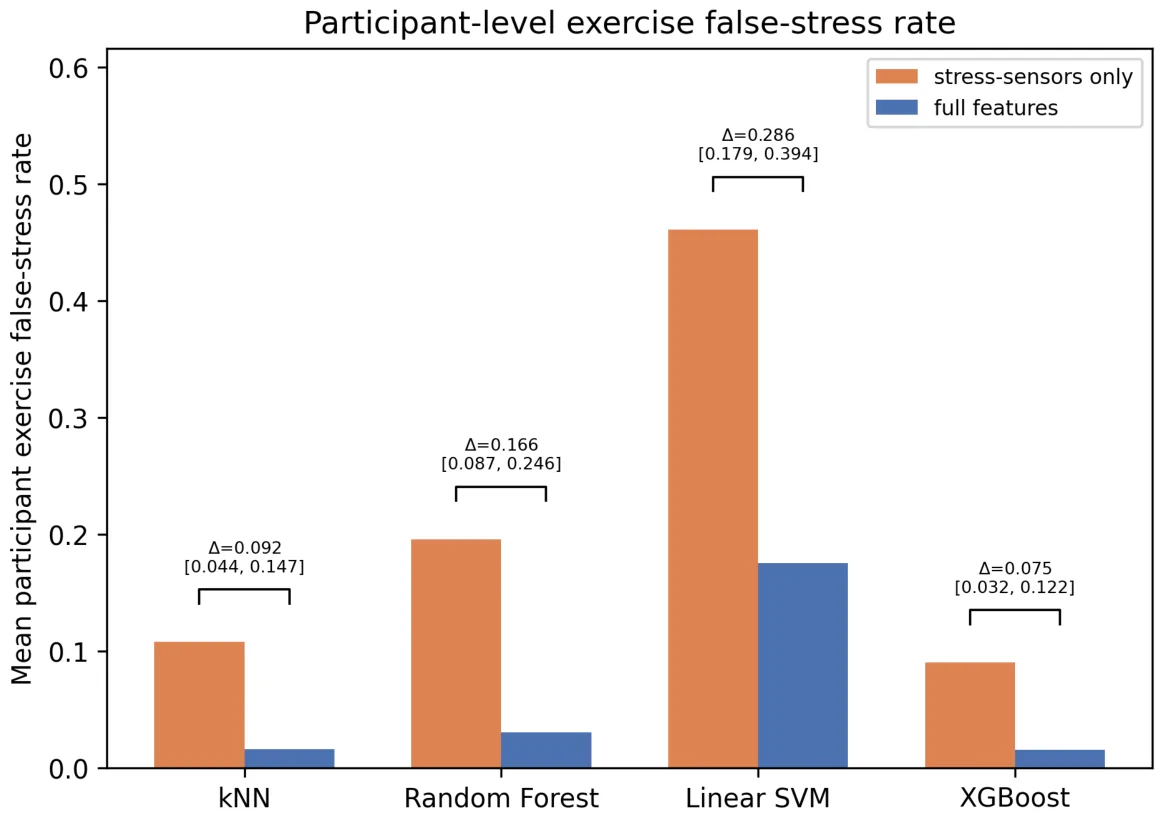
Participant-level exercise false-stress rate (FSER) under stress-sensors-only versus full features, all four models. Bars are unweighted means of the per-participant rates across the 31 participants with exercise windows. Each bracket reports the paired participant-level mean difference ΔFSER and its 95% participant-bootstrap interval, matching Table 13.

For every model, $\Delta \mathrm{FSER}={\mathrm{FSER}}_{\mathrm{stress}\text{-}\mathrm{sensors}}-{\mathrm{FSER}}_{\mathrm{full}\text{-}\mathrm{feature}}$ is computed on the same paired exercise windows scored under both feature sets (via the FSER-statistics script). Because exercise windows from the same subject are correlated, the primary uncertainty analysis is a participant-level bootstrap (5000 resamples of the 31 subjects who contribute exercise windows) together with a subject-level sign-flip permutation test on the 31 paired per-subject ΔFSER values; a window-level exact McNemar test is also reported but is explicitly not treated as independent-subject evidence, because it treats every exercise window as an independent trial despite within-subject correlation. Table 13 reports participant-level baseline/full FSER, absolute ΔFSER, relative reduction, the 95% bootstrap CI, and both test results. Figure 5 shows these participant-level rates and paired differences. Per-subject FSER values, comparison summaries, and bootstrap/McNemar outputs are provided as reproducibility-package CSV files.

All four participant-level 95% bootstrap CIs exclude zero, so the full-feature FSER reduction is statistically supported at the subject level for every model, not only descriptively. The window-level McNemar tests are reported in the reproducibility-package statistics outputs with exact *p*-values far below $10^{-100}$ for every model; these are not quoted as the primary evidence because windows from the same subject are temporally correlated and McNemar’s independence assumption is violated at the window level. The subject-level permutation test, which only uses one paired observation per subject, is the primary significance result and agrees with the bootstrap CIs. The phrase “paired statistics” in this manuscript refers specifically to this paired-window, subject-level-aggregated design. The four per-model tests are reported individually; all four permutation *p*-values (≤0.001) remain below a Bonferroni-corrected threshold of 0.05/4=0.0125.

Calibration of classifier probabilities and calibration of the agent’s rule-derived fused-confidence score are separate questions. Table 14 reports classifier calibration only, for the P1 stress-versus-exercise task. The evaluated score is the predicted probability of the positive class, stress, from the pooled out-of-fold LOSO predictions (10,824 windows, 36 held-out subjects). Probabilities are raw model outputs; no recalibration model was fitted, so no separate calibration split was needed. The Brier score is the binary mean squared difference between P(stress) and the stress indicator. The expected calibration error (ECE) uses 10 equal-width bins of P(stress), with the absolute difference between the observed stress fraction and the mean predicted probability weighted by the fraction of windows in each bin. Aggregation is at window level; 95% intervals come from a participant-cluster bootstrap (2000 resamples of the 36 subjects). The linear SVM produces no probability and is excluded. With full features, XGBoost had the lowest Brier score and kNN and XGBoost had the lowest ECE. RF had a low Brier score but a higher ECE. With stress-sensor-only features, the kNN and RF Brier scores exceeded the 0.167 score of a constant prediction at the stress prevalence (0.212). This analysis does not calibrate the agent’s fused-confidence score, which has no independently labelled reference outcome. Classifier calibration was not used to select, and does not justify, the 0.60, 0.45, or 0.65 agent parameters. The deployed Python agent uses the WESAD/PAMAP2 kNN vote share, whose calibration was not assessed.

### 6.5. Comparison with the Published PhysioNet Benchmark

Hongn et al. [25] report their own machine learning benchmark alongside the dataset: XGBoost with 10-fold cross-validation over an 80/20 train/test split and class-imbalance oversampling. Their samples were protocol segments rather than fixed windows: 154 stress and 102 rest segments after intra-participant normalization, and 120 one-minute aerobic segments versus 106 maximal-effort sprints. Whether the cross-validation folds were grouped by participant is not reported. Table 15 places that published benchmark next to the current RideSafe-AI LOSO results and states the validation-protocol difference explicitly, because the two are not run under the same protocol and a superiority claim would not be justified.

The published and current results differ across tasks and evaluation protocols. For example, the current P5 accuracy is 0.940 compared with the published 0.91, whereas the current P2 and P4 accuracies are lower. These values do not establish superiority because the splitting procedures, class handling, and evaluation conditions differ. The lower balanced accuracy and macro-F1 relative to raw accuracy in P2 and P4 indicate uneven performance across classes under the reported class imbalance. In P2, rest windows (25,648) outnumber stress windows (2299) by approximately 11:1. In P4, rest accounts for 70.3% of windows and anaerobic exercise for 1.5%. These distributions help explain why raw accuracy alone is insufficient, but the present comparison does not isolate the causal effect of any single design choice.

### 6.6. Agent Scenario Results

Table 16 tests the full decision pipeline on designed cases. These cases are not field data; they check whether the implementation produces the intended advisory state for normal, high-load, possible-stress, motion-compromised, invalid-input, and privacy-leakage inputs.

The full agent matched the expected safety status in 9/9 designed scenarios. This result supports the internal consistency of the agent logic under controlled inputs, but it is software-verification evidence rather than field validation.

### 6.7. Deterministic Scenario Verification Results

The implementation matched the specified safety status for all 500 deterministically generated windows, rejected all 20 privacy-attack windows, and withheld stress escalation in all 60 motion-compromised stress-label cases. The output distribution was 20 blocked, 20 guard-blocked, 60 critical watch, 70 high exertion, 60 motion-compromised watch, 80 normal, 70 possible stress, and 120 watch. These are conformance results on a deterministic verification suite. They do not estimate real-world accuracy, false-alert rates, or generalizability.

### 6.8. Ablation Results

Table 17 removes parts of the agent stack to show which components contribute to the designed-scenario behavior. The comparison is agent-level, so the main outcome is safety-status agreement rather than classifier accuracy alone.

Within the designed scenarios, separating exertion/load evidence, stress-label evidence, motion reliability, and privacy guarding increased agreement with the predefined expected outputs relative to the stress-only decision rule. This is a component-level verification result. It does not estimate changes in real-world false-alert rates or rider-stress classification accuracy.

### 6.9. Robustness and Privacy Results

The robustness suite achieved 18/18 expected checks. Missing HR summaries, missing stress features, out-of-range HR summaries, and short windows were blocked or downgraded rather than over-alerted. Raw streams, encoded signal fields, GPS traces, direct identifiers, long arrays, nested identifiers, and raw fields injected into bounded schemas were rejected by the guard. This is a targeted guardrail test, not a complete security audit.

### 6.10. Threshold Sensitivity Results

Only the alert-confidence threshold changed safety outputs (Table 18). On the nine designed scenarios, values of 0.55–0.80 matched all expected outputs, whereas 0.40 and 0.50 matched 7/9 because two watch-level cases became high_exertion. On the 500-window suite, 0.55 matched 485/500, 0.60–0.70 matched 500/500, and 0.80 matched 469/500. The 0.60 default therefore lies inside a plateau of agreement with the rule-derived expectations, but it is not uniquely supported by these inputs. Varying the abstention floor from 0.30 to 0.60, or the uncertainty boundary from 0.55 to 0.80, did not change any safety status. The abstention floor changed only the abstention flag and uncertainty category: the number of abstaining non-guard windows in the 500-window suite was 80 for floors of 0.30–0.50 and increased to 175 at 0.55 and 200 at 0.60. The uncertainty boundary changed only the low/moderate split. These results describe how the implemented rules respond to their parameters. They do not validate any of the three values against independent riding data.

### 6.11. Runtime Results

Table 19 and Table 20 separate reference-agent runtime from connected prototype measurements. This separation avoids treating Python benchmark timing as ESP32 firmware timing and separates a short bench INA219 power check from longer field-duration energy logging.

The current Python reference agent had millisecond-scale latency on the laptop host; its suitability for gateway-class devices such as a Raspberry Pi is plausible but was not tested. The Android bridge now contains a Java agent for live phone-side decisions and ESP32 comparison. ESP32-S3 firmware compilation after the embedded-agent and INA219 update reports 56% program storage use and 13% dynamic memory use for the C++ implementation, and the Android phone/watch modules compile after the same schema update. Controlled AP-mode checks showed the phone receiving Galaxy Watch summaries while connected to the ESP32 hotspot, the ESP32 gateway reporting a motion-reliable state, and the phone and ESP32 agents matching during a single live normal-state check and a 599.580 s repeated endpoint log. The repeated log measured 1058 phone–ESP32 endpoint requests with one timeout, p95 endpoint latencies of 71 ms for /status and 69 ms for successful /agent calls, and 528/528 successful phone–ESP32 agent-output matches. A subsequent bench instrumentation check added INA219 power telemetry to the ESP32-S3 firmware and observed approximately 4.90–4.91 V, 148.7–160.0 mA, and 724–740 mW while the MPU6500 motion gate remained motion_ok. The practical value of the dual-agent implementation is implementation traceability: the phone-side Java agent can bridge the wearable stream, present the rider/research interface, and log comparisons, while the ESP32-S3 C++ agent tests whether the bounded advisory logic (with a rule-based branch scorer) can run on a constrained bike-side node. Longer fixed-duration ride energy profiling remains future work. This supports the feasibility of migrating the decision pipeline to phone and ESP32 targets, but does not imply that Python, LLM reasoning, or large-model orchestration runs on the microcontroller.

### 6.12. Single-Participant Technical Feasibility Ride

**Figure 6:**
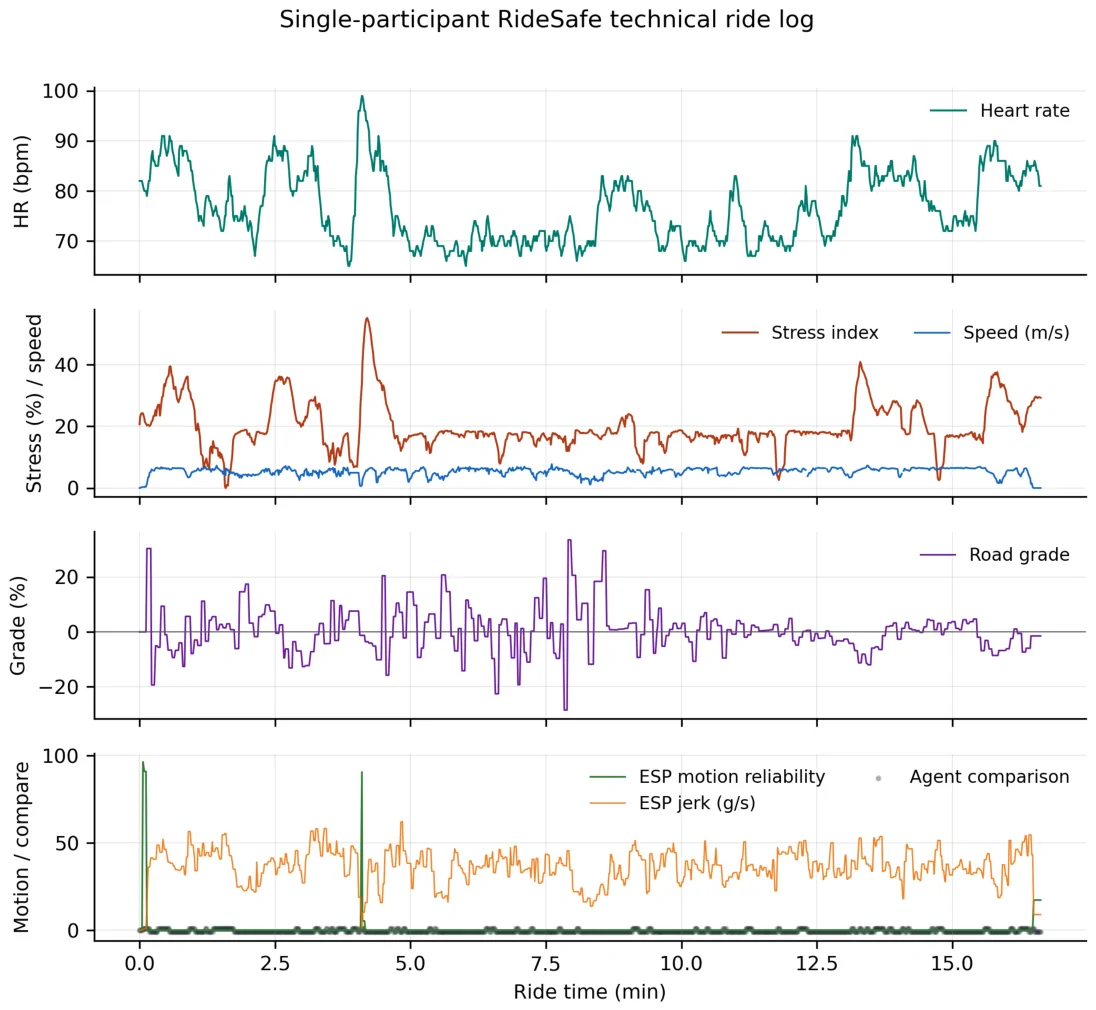
Time-series view of the single-participant technical ride log used in Table 21. The plot summarizes heart rate, stress-index output, phone speed, road-grade context, ESP32 motion reliability, ESP32 jerk, and phone–ESP32 agent comparison during the technical ride. The low ESP32 motion-reliability trace is an observed bike-frame/mounting and high-jerk feasibility finding, not a rider-stress result. The figure is included as implementation-feasibility evidence only and should not be interpreted as population-level rider-stress validation.

Two same-day single-participant technical feasibility rides were used as an implementation-only pilot to verify the wearable–phone–ESP32 pipeline during real riding, not to validate population-level rider stress detection; raw timestamps confirm these are two separate ride sessions roughly six hours apart on 18 July 2026, not one continuous ride split across files. Table 21 reports only automatically derived evidence from the primary, fully analyzed ride log and the completed ESP32 bench check. Figure 6 shows the time series of the primary ride. The other analyzed ride (14:09; 1067 rows over 18.53 min, 98.3% ESP32 connectivity) is slightly longer than the primary ride (20:14) and is summarized in the Single-User Engineering Validation Package below. During development on 17–18 July 2026, the same author also recorded further sessions with earlier app and firmware versions, including bench, stationary, and riding sessions. The analysis includes the two 18 July sessions recorded with the final 60-field logging schema, which contains the GPS-quality, segment-method, and manual-label fields required by the analysis script. An earlier riding session on the morning of 18 July (27.4 min) was recorded with a previous app version that lacks six of these fields; its aggregate summary is provided in the reproducibility package but is not analyzed here. The development sessions are not additional participants or independent validation data. The ride log contained 963 rows over 16.63 min, 100.0% GPS fill, 99.0% ESP32 connectivity, and INA219 power telemetry. Ride-specific HTTP p95 latency is not included in this table because endpoint timing is measured by the separate repeated endpoint log reported in Table 20.

The primary ride demonstrates that the prototype can record wearable, GPS, motion, power, and decision-output fields during the tested session. Phone–ESP32 agreement is an implementation diagnostic and provides no independent reference for the rider’s true stress state. Full-row agreement was 221/963 (22.9%). The 738 disagreement flags comprised 735 motion-only mismatches and three rows with a safety-status mismatch. Persistent high jerk from the ESP32 bike-frame IMU also produced 954 rough/mount-issue labels. These observations expose a mounting or calibration problem in the embedded motion gate. The firmware computes jerk as the sample-to-sample difference of unfiltered acceleration magnitude at 50 Hz and marks a frame window as compromised at a jerk RMS of 6 g/s or a dynamic RMS of 0.18 g. For broadband vibration, this jerk estimate scales approximately as $\sqrt{2}\;\sigma /\Delta t$, where σ is the dynamic RMS and Δt=0.02 s. The median dynamic RMS of 0.537 g in the primary ride predicts about 38 g/s, close to the logged median jerk RMS of 37.1 g/s. Ordinary frame vibration during riding therefore exceeds both limits, so a loose mount is not required to explain the saturation. This explanation is consistent with the firmware and logs but was not tested experimentally; low-pass filtering, recalibrated limits, and controlled mounting tests are required. They do not measure rider-stress accuracy. Likewise, the absence of stress-candidate rows cannot establish a low false-alert or missed-event rate without independently labelled reference events.

### 6.13. Single-User Engineering Validation Package

To separate completed implementation work from future multi-rider validation, an additional reproducible engineering-validation script was added. It consolidates the designed scenarios, expanded privacy/security perturbations, two single-user ride logs, the longest repeated phone–ESP32 endpoint log, and an advisory-output wording audit. This package is intentionally single-user and implementation-focused: it does not claim clinical diagnosis, real-traffic stress accuracy, accident reduction, deployment readiness, or multi-rider validation.

The package confirms 9/9 designed safety-state matches, 9/9 forbidden-key leakage checks, 18/18 expanded privacy/security perturbation checks, two 18 July 2026 ride logs with 2030 rows over 35.16 min and 98.6% ESP connectivity, a 1057/1058 successful endpoint-request audit, and 9/9 advisory labels free of clinical or accident-reduction claims. In the 14:09 ride, full-row phone–ESP32 agreement was 289/1067 (756 disagreements), and 14 rows met the heuristic load-context candidate rule; in the primary ride, no rows met it. These counts are implementation diagnostics without an independent stress reference. Full per-check outputs are retained in the reproducibility artefacts rather than repeated as another main-text table.

### 6.14. Research Question Summary

The current evidence answers the research questions within the stated architecture-feasibility boundary. RQ1 is answered as an implementation claim: RideSafe-AI separates privacy guarding, data quality, exertion/load modelling, public stress-label evidence, motion reliability, fusion, uncertainty/abstention, and advisory-state output into traceable steps. RQ2 is answered using two non-interchangeable evidence layers: the designed agent ablation improves from 3/9 safety-status agreement for the stress-only baseline to 9/9 for the full agent, while the official PhysioNet feature-set experiment provides subject-independent public evidence that adding HR and accelerometer summaries to stress-sensor features reduces exercise false-stress classification for all four tested models. The strongest PhysioNet result is full-feature XGBoost on P1 stress versus exercise: 0.963 accuracy, 0.944 macro-F1, and 0.015 exercise false-stress rate on 10,824 windows, with participant-level FSER bootstrap CIs excluding zero for every model. This is not a full bike-side pipeline validation. RQ3 is answered as a practical guardrail claim: raw-stream and identifier-style inputs are blocked in designed robustness tests, while the attribute-inference and reconstruction audits show that bounded summaries still carry residual information and therefore do not provide formal privacy. RQ4 is answered as feasibility evidence: Android Java and ESP32-S3 C++ implementations compile and run the logic with a rule-based branch scorer in place of the kNN models, with a 599.580 s endpoint log showing 1057/1058 successful endpoint calls and 528/528 successful normal/moderate-load phone–ESP32 agreement on safety-state, rider-state, and motion-gate outputs, without confirmed numerical agreement on intermediate confidence or uncertainty values. RQ5 is answered as logging readiness only: the ride schema captures GPS terrain and INA219 power telemetry needed to identify candidate false-stress escalations, but it does not yet prove causal reduction in real riders. RQ6 is answered as mismatch detection: the 18 July 2026 technical ride exposes motion-gate divergence caused by persistent ESP32 high-jerk readings, which is useful calibration evidence but not a validated uncertainty signal. Multi-rider validation, clinical validation, behavioral/safety-impact evaluation, field reconnect testing, and product-readiness hardening remain future work.

## 7. Discussion

The designed-scenario results show how the decision layer behaves relative to a stress-only rule under the specified inputs. Adding exertion/load evidence changes the rule-specified handling of physical load; adding motion reliability constrains stress-label escalation for high-motion inputs; and adding privacy guarding rejects the tested raw-stream payloads. The 500-window deterministic verification suite confirms the specified outputs for its generated cases. These results verify selected decision rules and do not estimate real-world false-alert rates or rider-stress accuracy. The LOSO baselines show that other classical models can improve public-dataset branch accuracy, especially on WESAD. The host resource profile does not favor kNN either: SVM was smaller and faster, and XGBoost offered a better PAMAP2 accuracy at sub-millisecond median latency. Because the Android and ESP32-S3 tiers currently run a rule-based scorer, the choice of an embedded classifier remains open and requires on-device measurement.

The designed scenarios test the decision logic for selected cases. They do not estimate real-world prevalence, classification performance, or safety effectiveness. The architectural evidence is that the prototype separates evidence sources, records decision reasons, and returns the rule-specified advisory states under the tested inputs.

This architectural framing is best understood relative to the closest prior systems rather than in isolation, and the comparison is one of design choices, not of outperforming any of them. A recent cycling-stress study combines wearable physiology with geo-referenced trips and open spatial data at a real-world scale this manuscript does not match [35], but it does not gate its output on motion reliability, does not enforce a data-minimizing boundary before the final decision, and is not demonstrated on constrained edge hardware; RideSafe-AI adds exactly these three mechanisms as separately traceable steps rather than claiming to exceed that study’s scale. Prior work has directly examined the separation of physical activity or physical stress from psychological stress using wearable physiological signals, including multimodal wearable models and, in Kaya et al., additional salivary cortisol information [20,21]. Because these studies use different populations, label structures, sensing configurations, and evaluation tasks, their point-performance values are not treated as directly comparable with the PhysioNet experiment reported here. RideSafe-AI instead uses the PhysioNet experiment as complementary evidence for the value of explicitly representing exertion context when interpreting stress-labelled physiological patterns. TinyML precedents already establish that wearable stress inference can run on constrained microcontrollers [62,63]; relative to these, RideSafe-AI contributes the combination of motion-reliability gating, bounded/schema-guarded data exchange, conservative fusion with abstention, and corresponding platform-specific implementations that share processing stages and an output vocabulary. This wording does not imply numerical equivalence across Python, Android, and ESP32-S3. None of these five systems is claimed to be surpassed; the contribution being compared is the architectural integration itself, not a new classifier, a new signal-processing method, or a new privacy mechanism.

The architecture also supports a practical deployment split. The ESP32-S3 implementation is well suited for bike-side sensing, motion-window extraction, local decision logic, compact advisory-state signalling, and short-window power telemetry. The Android phone is better suited for the Java live bridge, user interface, ESP32 comparison, and detailed logs, while a Raspberry Pi or similar gateway-class device remains better suited for Python reference logging and longer comparison reports. This layered split is consistent with edge-intelligence designs that distribute sensing, inference, and coordination across heterogeneous edge nodes under latency, energy, and privacy constraints [65,66,67]. Comparing Android and ESP32-S3 outputs can reveal implementation or sensor-gating mismatches. The 528/528 categorical agreement result comes from an approximately 10 min AP-mode endpoint session limited to its observed normal/moderate-load inputs. The primary technical ride is a different evaluation and produced 221/963 full-row matches, chiefly because the watch and bike-frame motion gates diverged. These findings should therefore be read together rather than as general equivalence evidence. A stronger publication claim would require on-device, scenario-wise platform comparison across normal, high-exertion, possible-stress, motion-compromised, blocked-input, and privacy-guard cases, including safety-state agreement, rider-state agreement, motion-gate agreement, confidence or uncertainty deltas, latency, timeout/reconnect behavior, mismatch analysis, and fixed-duration field energy profiling.

The supported claims are architecture feasibility, public-dataset branch modelling, protocol-reconstructed PhysioNet stress/exercise feature-set evidence, decision-logic verification, tested privacy-guard behavior, and short-session Android/ESP32 feasibility. The unsupported claims are equally important: the paper does not validate real-world rider-stress detection, clinical diagnosis, safety or behavioral impact, long-duration deployment stability, or production security. The current evidence is therefore suitable for an architecture-feasibility submission, not for a deployment-readiness or safety-effectiveness claim.

## 8. Privacy Design

The privacy design is based on data minimization at the agent boundary. The final reasoning layer receives bounded summaries rather than raw physiological streams. The Python reference applies input/output field guards; the ESP32-S3 guards incoming fields; Android constructs bounded inputs internally but does not apply an equivalent guard to its inputs, outputs, or logs. This is a practical privacy boundary for the current prototype, not a formal differential-privacy guarantee.

The additional reproducibility audit expands the privacy evidence while preserving this boundary. Table 22 separates direct guard blocking, residual attribute inference from bounded summaries, empirical raw-sequence reconstruction, and non-unique inversion of bounded feature summaries. The direct guard tests blocked 31/31 attempted raw-stream, identifier, encoded, nested, and long-array payloads across the robustness and bounded-benchmark artefacts. A separate WESAD attribute-inference check deliberately assumed that an attacker can see bounded summary features and tries to infer the withheld stress condition. Under LOSO validation, a random-forest attacker reached 0.896 accuracy, 0.883 balanced accuracy, and 0.885 macro-F1, compared with a 0.646 class-prior baseline. The inferred attribute is the same WESAD condition label that the stress-label branch is designed to output, so this test shows that the bounded features carry that intended information. Inference of attributes the system is not designed to output, such as identity or demographic characteristics, was not tested.

The reconstruction threat model assumes that an attacker can observe six bounded per-window HR summaries exposed by the feature pipeline: hr_mean, hr_std, hr_min, hr_max, hr_range, and hr_slope. The attacker also knows the 10 s window duration and 1 Hz Empatica E4 HR sample rate, but does not receive the raw HR sequence, subject identity, or other feature families. One constrained-optimization attack was evaluated on 120 windows pooled across subjects. A separate learned-attack benchmark used 28 subjects to form a 2499-window training library and evaluated 400 windows from eight held-out subjects. That benchmark includes constant/mean, nearest-neighbour, ridge, random-forest, and XGBoost reconstruction methods. Because the optimization and learned-attack evaluations use different samples and splitting procedures, their metric values are reported separately and are not used for direct attacker ranking. Within the common 400-window subject-wise evaluation, the learned methods improve on the constant/mean baseline, showing that the permitted summaries retain reconstructive information. The size of this gain should be read against the baseline: repeating the window mean already gives an RMSE below 1 bpm for 50.5% of the 10 s windows, and the median per-window $R^{2}$ is negative for every method (for example, −2.59 for random forest). The summaries therefore reveal the level and range of heart rate closely, but the within-window time course is recovered only partly. These six PhysioNet HR summaries are more detailed than the heart-rate fields of the agent input schema (mean heart rate and heart-rate drift), so the test describes the feature pipeline rather than the narrower agent boundary. These empirical attacks demonstrate residual disclosure risk; they do not provide a formal upper bound on leakage or certify resistance to stronger attackers.

For future recruited-rider validation, the privacy boundary must be embedded in a broader GDPR governance process before any new participant sensing begins. That process should define the lawful basis and explicit consent approach for physiological sensing, purpose limitation, data minimization, retention and deletion periods, pseudonymization, access control, secure transport, audit logging, participant rights handling, ethics approval, and a DPIA-style risk assessment. These governance requirements are not presented as completed field-study controls in the current manuscript because the two 18 July 2026 technical rides were performed by one of the authors as engineering self-testing and are reported only as implementation-feasibility evidence, not as a recruited human-subject validation dataset.

The current threat model covers raw physiological stream leakage, direct identifiers, network interception, compromised local devices, adversarial inputs, and GDPR governance. Implemented controls include bounded-input construction on all tiers, Python input/output field guards, an ESP32-S3 input-field guard, and constrained final outputs; Android has no equivalent schema guard. Residual risks remain for renamed or encoded streams, authenticated transport, replay protection, endpoint hardening, log minimization, access control, retention/deletion policy, and a larger adversarial privacy test suite before any recruited-rider deployment.

## 9. Validation Roadmap

The current manuscript intentionally stops at architecture and technical feasibility evaluation. It includes a single-participant implementation log, but a full field-validation claim would require a separate recruited rider-context study with ethics/GDPR approval. That validation should include recruited riders, informed consent, controlled and real-road routes, synchronized wearable and bike-frame sensing, route context such as slope and surface quality, rider self-report or event markers, and predefined evaluation metrics for rider-state agreement, false alerts, missed alerts, latency, packet loss, reconnection behavior, and energy use.

The two same-day technical rides by one participant provide an intermediate engineering self-test; the primary 16.63 min ride is analyzed in Table 21. Single-subject and self-tracked health-data literature supports the idea that dense longitudinal data from one person can be useful for individual-level technical or methodological questions, provided that the interpretation does not become a population-level claim [83]. Reporting should also remain transparent about design, setting, measurements, bias, and limitations, consistent with observational-reporting principles such as STROBE [84]. In RideSafe-AI, the 18 July 2026 log strengthens the engineering evidence by verifying automatic wearable–phone–ESP32 logging, bounded feature generation, phone–ESP32 comparison, GPS terrain enrichment, motion-gate behavior, and INA219 energy telemetry during a real ride. This evidence is labelled as single-participant feasibility evidence only, with broader validation handled in the roadmap.

The same boundary applies to the current ride table: it is a technical pipeline check rather than a validation cohort. The useful evidence is end-to-end connectivity during real riding, synchronized wearable/phone/ESP32 logs, automatic ride-segment labels, motion-gate and privacy-guard behavior, phone–ESP32 agreement on outputs, GPS quality checks, and battery or direct-energy observations.

A larger next empirical step is a controlled pilot with approximately 10–20 riders and repeated route segments covering rest, normal cycling, increased exertion, rough-surface motion, recovery, and, where ethically appropriate, stress-inducing or time-pressure proxy conditions. This would directly test the central ambiguity addressed by RideSafe-AI: elevated physiological load caused by exertion should be separated from public stress-label evidence, and high motion should reduce confidence in rider-facing escalation. The pilot should include held-out-rider testing rather than only window-level random splits, because repeated windows from the same rider are not independent.

Before that recruited pilot, the implementation roadmap includes an external-data preparation phase that is now represented in code and generated outputs. The local derived PhysioNet support file remains a useful negative audit because it contains 3425 bounded stress-labelled windows without exercise/rest labels. The official PhysioNet v1.0.1 folders, however, have now been reprocessed from event-tagged STRESS, AEROBIC, and ANAEROBIC protocols into 36,472 protocol-labelled windows and used for stress-versus-exercise LOSO and FSER-style feature-set comparisons. The GiroE adapter prepares 3336 e-bike support windows for contextual feature checks, with heart-rate and speed coverage in the current derived file but no validated stress labels and no populated grade or accelerometer summaries. The cycling-stress and e-scooter study records provide protocol variables and scenario labels in the support registry rather than trained model evidence. The resulting fixed RideSafe-AI data-collection schema for the author’s own rides includes synchronized smartwatch summaries, Android GPS and battery logs, ESP32/MPU6500 motion reliability, INA219 power telemetry, phone-agent output, ESP32-agent output, route segment labels, and short rider self-report markers for perceived exertion, perceived stress, road roughness, and traffic pressure. This schema is intended to make the later ethics/GDPR-approved data collection directly comparable with public-data evidence while preserving the data-minimizing design.

The immediate roadmap is therefore concrete: repeat the technical ride after mount/threshold calibration, run multi-scenario live phone–ESP32 agreement tests, complete fixed-duration energy and reconnect logging, and then perform an ethics/GDPR-approved controlled rider-context pilot with held-out-rider evaluation. That pilot should report macro-F1, balanced accuracy, Matthews correlation coefficient, AUROC/AUPRC where labels support them, calibration, false-alert/missed-alert rates, latency, packet loss, reconnection behavior, energy use, and rider-facing human-factors outcomes.

## 10. Limitations

This study has the following limitations:

1. The scenario evaluation uses designed bounded inputs rather than a large real ride-context dataset.
2. PAMAP2 provides physical activity and heart-rate context but not psychological stress labels; newer stress–exercise, cycling-stress, e-bike, and e-scooter sources are now represented by an implemented external-support registry. Official PhysioNet protocol-window modelling is completed as a same-observation feature-set experiment (stress-sensor-only versus full features, Section 5.3) that provides subject-independent public stress/exercise evidence, not a run of the RideSafe-AI agent itself and not a real e-bike/e-scooter rider trial; GiroE contains e-bike context windows without stress ground truth. Neither source is claimed as completed RideSafe-AI rider validation.
3. WESAD provides stress labels but is not an electric micro-mobility traffic dataset.
4. The current agent ablation is scenario-based; the PhysioNet feature-set result is empirical but does not run the complete bike-side agent.
5. GPS terrain and power telemetry are logged and used for candidate context checks, but the present technical rides do not prove causal reduction in real rider false-stress escalation.
6. The motion-reliability gate is evaluated as a confidence-control mechanism. In the 18 July 2026 technical ride, the ESP32 motion gate was saturated by persistent high-jerk readings (954/963 rough/mount-issue labels and 735 motion-only phone–ESP32 mismatches), so mount/threshold calibration and re-testing are required before claiming field-calibrated motion discrimination. The unfiltered finite-difference jerk estimate and the 6 g/s limit are a likely cause (Section 6.12).
7. The privacy boundary is data minimization and guardrail enforcement, not differential privacy, non-reconstruction, or immunity to attribute inference.
8. The ESP32-S3 implementation is a bounded embedded C++ agent, not a Python or LLM runtime; the current live checks cover a normal-state connected path and one approximately 10 min repeated endpoint log, but multi-scenario live agreement, field reconnect behavior, and longer-duration stability remain future work.
9. The three tiers share uncertainty rules, safety mapping, and threshold constants in source code, but one rider-state precedence rule, their branch scorers, and their motion inputs differ, and the Android tier has no privacy guard. A host-level matched-input test showed identical Android and ESP32-S3 decision outputs and isolated the single Python precedence difference, but no test was run on the devices themselves (Section 3.8). Numerical equivalence of intermediate confidences is therefore not claimed. In live Android operation, the BVP, EDA, temperature, speed-drop, and cadence-drop inputs are generated from heart-rate-based proxies, so the technical rides did not exercise the WESAD-trained stress-label model.
10. The alert-confidence threshold (0.60), fused-confidence abstention floor (0.45), and moderate-uncertainty boundary (0.65) are heuristic prototype parameters. All three were varied in a software sensitivity check on rule-derived scenarios; only the alert threshold changed safety outputs. None of the three values was optimized or validated against independent labelled riding data.
11. Model-wise resource measurements were made on a laptop host. None of the four classifiers is deployed on the Android or ESP32-S3 tiers, which use a rule-based scorer, and energy per inference was not measured.
12. Reference/gateway-side latency, phone-observed ESP32 endpoint latency, and a short INA219 bench power check are measured, but field-duration ESP32 energy logs still require a fixed-duration connected ESP32/phone/watch measurement session.
13. Safety or behavioral impact is not yet evaluated with human-factors outcomes such as distraction, trust, false-alarm tolerance, or rider response.
14. The included two same-day single-participant technical feasibility rides strengthen implementation evidence; broader rider-context validation remains future work.
15. Real rider validation requires ethics/GDPR approval.

## 11. Conclusions

RideSafe-AI demonstrates a data-minimizing, motion-gated decision-support architecture for non-clinical electric micro-mobility rider-context monitoring. The prototype separates exertion/load proxy estimation, public-dataset stress-label classification, motion reliability, data quality, platform-specific privacy guarding, deterministic fusion, rule-based uncertainty categorization, abstention, and advisory-state generation. PAMAP2 and WESAD support their specified public-dataset branch tasks. The protocol-reconstructed PhysioNet experiment provides subject-wise laboratory evidence that adding heart-rate and accelerometer summaries reduces exercise false-stress classification relative to stress-sensor-only features. The deterministic 500-window software-verification suite checks selected decision rules, and two same-day technical rides by one participant demonstrate integration and logging. The primary ride also exposes an ESP32 motion-gate calibration problem. These findings support architecture feasibility; they do not establish real-world rider-stress accuracy, safety impact, cross-platform equivalence, or performance across riders and road conditions.

## Figures and Tables

**Table 1:** Comparison of RideSafe-AI against the closest prior studies discussed in Related Work, based on features explicitly reported in each cited study. ✓: feature explicitly present; ×: the cited source indicates that the feature is not part of the reported architecture or method; △: a related mechanism is present but is architecturally different (e.g., offline signal denoising or dataset-level curation rather than a runtime decision gate); NR: feature could not be established from the cited source and should not be interpreted as absent; N/A: not applicable to the study’s design. Feature columns: Str. = stress evidence; Ext. = exertion/activity evidence; Mob. = cycling/e-mobility context; MGate = active motion-reliability decision gate; DQGate = runtime data-quality gate; Abst. = abstention/reject outcome; DMin = data-minimizing output boundary; Edge = microcontroller/TinyML deployment; MTier = independently cross-validated multi-tier deployment. RideSafe-AI is marked △ for Edge because the microcontroller runs the decision pipeline with a rule-based scorer rather than a trained classifier, and △ for MTier because its three tiers share processing stages and output categories but have not been shown to be equivalent (Section 3.8). This table compares reported architectural features only, not cross-study predictive performance.

Study	Str.	Ext.	Mob.	MGate	DQGate	Abst.	DMin	Edge	MTier
Kaya et al. [20]	✓	✓	×	×	×	×	×	×	×
Abdel-Latif et al. [21]	✓	✓	×	×	△	×	×	×	×
Moser et al. [35]	✓	×	✓	×	NR	×	×	×	×
Ganesan et al. [62]	✓	×	×	×	△	△	△	✓	×
Abu-Samah et al. [63]	✓	×	×	×	×	×	△	✓	×
Hongn et al. [25]	✓	✓	△	×	△	×	△	×	×
Pejhan et al. [37]	△	×	✓	N/A	N/A	N/A	×	NR	N/A
GiroE [36]	×	△	✓	×	NR	N/A	×	N/A	×
RideSafe-AI (this work)	✓	✓	✓	✓	✓	✓	✓	△	△

**Table 2:** Decision thresholds confirmed by source inspection in the Python reference, Android, and ESP32-S3 implementations. Identical constants do not imply identical outputs, because the branch confidences compared with these thresholds come from kNN models in Python and from a rule-based scorer on Android and ESP32-S3.

Threshold	Value	Role	Confirmed on
Alert-confidence threshold	0.60	A high exertion or stress label is treated as confident enough to drive escalation only at or above this value	Python, Android, ESP32-S3
Fused-confidence abstention floor	0.45	Below this value, the abstention flag is set and uncertainty is reported as high; the safety status is not changed by this rule	Python, Android, ESP32-S3
Moderate-uncertainty boundary	0.65	Below this value (and above the abstention floor), uncertainty is reported as moderate rather than low	Python, Android, ESP32-S3

**Table 3:** Mapping from the intermediate rider-state assessment to the final restricted safety-status output.

Condition (Rider State or Gate)	Safety Status
Data quality poor (fusion not reached)	blocked
Forbidden/raw field detected (privacy guard triggers before fusion)	blocked_by_guard
Motion-compromised and stress label high (overrides rider state below)	motion_compromised_watch
combined_high_load	critical_watch
high_physical_load	high_exertion
possible_stress	possible_stress
uncertain_load_watch, uncertain_stress_watch, or moderate_physical_load	watch
normal_or_low_load (default)	normal

**Table 4:** Implementation comparison obtained by source inspection of the archived code (OSF reproducibility package). “Same” means the same rule and constants in source code; it does not mean that identical outputs were demonstrated.

Component	Python Reference	Android (Java)	ESP32-S3 (C++)
Physiological inputs	Bounded feature windows from public datasets or designed scenarios	Watch HR, acceleration, and gyroscope; BVP/EDA/temperature and speed/cadence-drop inputs generated from HR-based proxies	Aggregate inputs received from the phone
Motion input	Summary fields in the input schema	ESP32-S3 frame summaries when fresh; otherwise watch proxy	MPU6500, 50 Hz, 2 s windows
Exertion and stress branches	Weighted kNN (k=15 PAMAP2; k=11 WESAD)	Rule-based scorer	Rule-based scorer (same as Android)
Motion rule and multipliers	Same	Same	Same, plus a frame-window gate
Rider-state fusion	Medium exertion checked after the stress-label rules	Medium exertion checked before the stress-label rules	Same as Android
Uncertainty rules, safety mapping, thresholds	Same	Same	Same
Privacy guard	Input/output key guard	Not implemented	Input key guard; returns blocked_by_guard
Matched-input test	Performed on a host computer with the extracted decision code (see text); not performed on the devices		

**Table 5:** Model-wise host resource profile under identical conditions (same features, LOSO folds, and hyperparameters as the LOSO baseline comparison in Section 6.1). Stored size is the median serialized estimator size over folds. Latency is single-window predict time. Energy per inference was not measured. These are host measurements, not ESP32-S3 or Android measurements; none of the four classifiers is deployed on those tiers.

Dataset	Model	Pooled LOSO acc.	Stored Size (kB)	Fit Time/ Fold (s)	Latency Median (ms)	Latency p95 (ms)	Peak Memory Increase (MiB)
PAMAP2	kNN	0.821	105	0.005	1.27	2.13	1.1
	RF	0.809	10,803	1.48	38.5	51.9	34.3
	SVM	0.791	55	0.038	0.26	0.72	4.9
	XGBoost	0.857	480	0.218	0.66	1.19	6.7
WESAD	kNN	0.835	122	0.002	0.65	1.94	0.8
	RF	0.896	2416	1.10	37.8	51.8	10.7
	SVM	0.891	48	0.011	0.23	0.47	1.9
	XGBoost	0.892	168	0.119	0.83	1.60	4.9

**Table 6:** Held-out public-dataset model evidence used by RideSafe-AI. Accuracy intervals are 95% Wilson confidence intervals.

Branch	Dataset	Model	Split	Train	Test	Accuracy (95% CI)	Macro-F1	Evidence Boundary
Exertion/load	PAMAP2	weighted kNN, k = 15	random held-out	1378	591	0.816 (0.782–0.845)	0.806	Physical exertion/load proxy; not psychological stress
Stress-label	WESAD	weighted kNN, k = 11	subject-wise held-out	650	233	0.850 (0.798–0.890)	0.823	Laboratory condition-label classification; not rider-stress detection accuracy

**Table 7:** Held-out class-level precision, recall, and F1 for the current weighted kNN branch models.

Branch	Class	Precision	Recall	F1	Support
Exertion/load	high	0.871	0.693	0.772	127
Exertion/load	low	0.877	0.762	0.815	168
Exertion/load	medium	0.773	0.899	0.831	296
Stress	high	0.900	0.651	0.755	83
Stress	low	0.832	0.960	0.892	150

**Table 8:** Pooled LOSO confusion matrices for the current weighted kNN branch models. Rows are true labels and columns are predicted labels. PAMAP2 has three exertion classes; WESAD has two stress-label classes.

Branch	True Label	Pred. High	Pred. Low	Pred. Medium
Exertion/load	high	314	1	111
Exertion/load	low	0	451	107
Exertion/load	medium	32	101	852
Stress	high	215	98	–
Stress	low	48	522	–

**Table 9:** Subject-wise LOSO validation and baseline comparison. Pooled accuracy intervals are 95% Wilson intervals; fold intervals are 95% confidence intervals over subject folds.

Branch	Dataset	Model	Pooled acc. (95% CI)	Fold acc. Mean (95% CI)	Fold Macro-F1 Mean (95% CI)
Exertion/load	PAMAP2	kNN	0.821 (0.804–0.838)	0.840 (0.783–0.897)	0.758 (0.630–0.885)
Exertion/load	PAMAP2	RF	0.809 (0.791–0.826)	0.829 (0.759–0.900)	0.752 (0.619–0.884)
Exertion/load	PAMAP2	SVM	0.791 (0.772–0.808)	0.811 (0.748–0.873)	0.741 (0.620–0.861)
Exertion/load	PAMAP2	XGBoost	0.857 (0.841–0.872)	0.872 (0.825–0.918)	0.792 (0.656–0.928)
Stress-label	WESAD	kNN	0.835 (0.809–0.858)	0.834 (0.800–0.868)	0.807 (0.769–0.846)
Stress-label	WESAD	RF	0.896 (0.874–0.914)	0.895 (0.836–0.954)	0.873 (0.791–0.956)
Stress-label	WESAD	SVM	0.891 (0.869–0.910)	0.891 (0.844–0.938)	0.878 (0.824–0.932)
Stress-label	WESAD	XGBoost	0.892 (0.870–0.911)	0.892 (0.825–0.958)	0.871 (0.783–0.958)

**Table 10:** Subject-wise LOSO classification results on reconstructed PhysioNet laboratory protocol windows. These experiments use available wearable features; bike-frame motion, GPS terrain, and INA219 power inputs are absent. They do not evaluate the complete RideSafe-AI pipeline or validate rider-state assessment in real traffic. Exercise false-stress rates in this table are pooled window-level rates.

Experiment	Best or Reported Model	Windows	Accuracy	Balanced Accuracy	Macro-F1	Extra Result
P1 stress versus exercise	XGBoost, full features	10,824	0.963	0.934	0.944	Exercise false-stress rate 0.015
P1 stress versus exercise	RF, full features	10,824	0.958	0.943	0.938	Exercise false-stress rate 0.031
P2 stress versus rest/non-task	RF, full features	27,947	0.879	0.610	0.608	Rest-dominant protocol gaps; not rider rest validation
P3 stress versus aerobic versus anaerobic	RF, full features	10,824	0.923	0.776	0.792	Anaerobic class has 554 windows
P4 stress/rest/aerobic/ anaerobic	XGBoost, full features	36,472	0.754	0.405	0.425	RF/SVM were runtime-limited for this largest task
P5 aerobic versus anaerobic	RF, full features	8525	0.940	0.715	0.733	Anaerobic is 6.5% of P5 windows (554/8525)

**Table 11:** PhysioNet participant contribution audit across protocols. Two published-benchmark cross-check values (aerobic, anaerobic) are shown for comparison with Hongn et al. [25].

Category	Local	Published
Total unique subjects	36	36
Subjects with stress data	34	36
Subjects with aerobic data	30	30
Subjects with anaerobic data	31	31
Subjects with stress and any exercise	29	–
Subjects with stress and aerobic	28	–
Subjects with stress and anaerobic	29	–
Subjects with all four P4 classes	28	–
P1 held-out folds with both classes	29/36	–
P1 held-out folds, single class only	7/36	–

**Table 12:** P1 sensitivity check: all 36 subjects (headline) versus the 29 dual-condition subjects only (both stress and exercise present, retrained end-to-end). Full feature set. FSER is the pooled window-level rate; Table 13 reports participant-level means, which differ slightly.

Model	Subjects	Accuracy	Balanced Accuracy	Macro-F1	FSER
XGBoost	36	0.963	0.934	0.944	0.015
XGBoost	29 dual	0.962	0.928	0.938	0.016
RF	36	0.958	0.943	0.938	0.031
RF	29 dual	0.964	0.940	0.943	0.021
kNN	36	0.932	0.862	0.890	0.017
kNN	29 dual	0.932	0.850	0.881	0.016
Linear SVM	36	0.837	0.865	0.796	0.184
Linear SVM	29 dual	0.826	0.856	0.777	0.193

**Table 13:** FSER statistics for the P1 stress-sensors-only versus full-feature comparison (31 subjects with exercise windows, 8525 paired exercise windows). ΔFSER and its 95% CI use a participant-level bootstrap; the permutation *p*-value is a subject-level sign-flip test on paired per-subject ΔFSER; the McNemar *p*-value is window-level and carries the independence caveat below. FSER values are participant-level means (mean of per-subject rates), which differ slightly from the pooled window-level rates in Table 10 and Table 12. With 5000 sign flips, <0.0002 denotes that no permuted statistic reached the observed value.

Model	FSER Stress-Sensors	FSER Full	ΔFSER	Relative Reduction	ΔFSER 95% CI	Permutation *p*	McNemar *p* (Window-Level)
XGBoost	0.090	0.015	0.075	83.1%	[0.032, 0.122]	0.0010	<$10^{-100}$
RF	0.196	0.030	0.166	84.6%	[0.087, 0.246]	<0.0002	<$10^{-100}$
kNN	0.108	0.016	0.092	85.3%	[0.044, 0.147]	0.0002	<$10^{-100}$
Linear SVM	0.461	0.175	0.286	62.0%	[0.179, 0.394]	<0.0002	<$10^{-100}$

**Table 14:** Probability calibration of P1 stress-versus-exercise classifiers (pooled out-of-fold LOSO predictions; positive class = stress; raw probabilities, no recalibration). Values in brackets are 95% participant-cluster bootstrap intervals. This is classifier calibration, not calibration of the agent’s fused-confidence score.

Features	Model	Brier Score	ECE (10 bins)
Full	kNN	0.055 [0.040, 0.072]	0.028 [0.021, 0.049]
	RF	0.039 [0.028, 0.052]	0.081 [0.059, 0.111]
	XGBoost	0.030 [0.021, 0.040]	0.029 [0.023, 0.045]
Stress sensors only	kNN	0.183 [0.155, 0.212]	0.125 [0.085, 0.166]
	RF	0.191 [0.149, 0.239]	0.122 [0.069, 0.188]
	XGBoost	0.153 [0.126, 0.185]	0.053 [0.035, 0.105]

**Table 15:** Published PhysioNet benchmark [25] versus current RideSafe-AI LOSO results. Validation protocols differ, so this is not a same-protocol superiority comparison.

Task	Published PhysioNet Result (XGBoost)	RideSafe-AI Current Result	Validation Difference
Stress vs. rest (P2)	Accuracy 0.93, F1 0.92	RF, full features: accuracy 0.879, balanced accuracy 0.610, macro-F1 0.608 (27,947 windows)	Published: 256 stress/rest protocol segments, 10-fold CV over an 80/20 split with oversampling; participant grouping not reported. RideSafe: subject-wise LOSO, no oversampling, natural 8.2%/91.8% stress/rest class imbalance.
Aerobic vs. anaerobic (P5)	Accuracy 0.91, F1 0.91	RF, full features: accuracy 0.940, balanced accuracy 0.715, macro-F1 0.733 (8525 windows)	Published: 120 one-minute aerobic segments versus 106 sprints, 10-fold CV over an 80/20 split with oversampling; participant grouping not reported. RideSafe: subject-wise LOSO, natural class imbalance (554/8525 = 6.5% anaerobic), 31 total exercise-protocol subjects.
Four-class stress/rest/aerobic/anaerobic (P4)	Accuracy 0.84, F1 0.84	XGBoost, full features: accuracy 0.754, balanced accuracy 0.405, macro-F1 0.425 (36,472 windows)	Published: protocol segments (rest, stress, aerobic, sprint), 10-fold CV over an 80/20 split with oversampling; participant grouping not reported. RideSafe: subject-wise LOSO, no oversampling; rest is 70.3% of windows and anaerobic only 1.5%.

**Table 16:** Designed scenario results for the full RideSafe-AI agent. Human-readable labels are used in the table; implementation state identifiers use snake case.

Scenario	Expected	Observed	Quality	Motion Gate	Confidence
Normal low load	normal	normal	good	motion ok	0.664
Moderate physical load	watch	watch	good	motion ok	0.504
High physical load	high exertion	high exertion	good	motion ok	0.664
Possible stress, clean motion	possible stress	possible stress	good	motion ok	0.647
Combined high load	critical watch	critical watch	good	motion ok	0.813
Motion-compromised stress	motion-compromised watch	motion-compromised watch	good	motion compromised	0.480
Short window	watch	watch	warning	motion ok	0.504
Out-of-range HR	blocked	blocked	poor	blocked	0.000
Raw-stream leakage	blocked by guard	blocked by guard	–	–	–

**Table 17:** Ablation results on the designed scenario benchmark.

Variant	Agreement	Privacy Blocked	Mean Runtime (ms)
Stress-only	3/9	0	2.219
Stress + exertion/load	7/9	0	4.138
Stress + exertion/load + motion	8/9	0	4.137
Full RideSafe-AI agent	9/9	1	3.893

**Table 18:** Software sensitivity of the three heuristic parameters in the Python reference agent (one parameter varied at a time; others at default). Agreement is with rule-derived expected safety statuses, not with independent labels. Abstentions exclude the 20 guard-blocked windows.

Parameter	Values	9 Scenarios	500-Window Suite	Abstentions (500)
Alert confidence	0.40, 0.50	7/9	420/500	80
	0.55	9/9	485/500	80
	0.60 (default), 0.65, 0.70	9/9	500/500	80
	0.80	9/9	469/500	80
Abstention floor	0.30–0.50 (default 0.45)	9/9	500/500	80
	0.55; 0.60	9/9	500/500	175; 200
Uncertainty boundary	0.55–0.80 (default 0.65)	9/9	500/500	80

**Table 19:** Runtime evidence for the current Python reference agent. Times are in milliseconds; Bytes is the mean output JSON size. Values are taken from the archived output files of the reproducibility package; wall-clock timings vary between runs.

Evaluation	Runs	Mean	P50	P95	Min	Max	Bytes
Repeated benchmark	100	4.834	4.599	6.427	3.748	8.735	2950.1
Scenario evaluation	9	3.547	–	–	0.408	5.735	–
Robustness evaluation	18	1.108	–	–	0.054	5.074	–
Large bounded benchmark	500	2.239	–	–	0.035	6.510	–

**Table 20:** Endpoint latency, availability, and energy-measurement status for the current prototype.

Measurement	Current Value	Interpretation
Python reference/gateway-side agent latency	100 runs: mean 4.834 ms, p50 4.599 ms, p95 6.427 ms, min 3.748 ms, max 8.735 ms	Reference benchmark only; not live ESP32 endpoint latency.
Android phone, Watch, and ESP32 build/deploy	Gradle assembleDebug completed for phone/watch; archived ESP32-S3 compile logs report approximately 744 kB (56%) program storage and approximately 45.2 kB (13%) dynamic memory after the bounded-agent and INA219 updates; AP-mode firmware served its local web interface at IP address 192.168.4.1.	Confirms deployable prototype components and that the C++ pipeline with motion and power telemetry fits on ESP32-S3; exact byte counts depend on the compiled sketch revision.
Repeated phone–ESP32 endpoint log	529 ticks over 599.580 s produced 1058 endpoint requests: 529/529 /status successes and 528/529 /agent successes, with 1 SocketTimeoutException; endpoint failure rate was 1/1058 (0.095%).	Provides a repeated connected live endpoint log from the phone while connected to the ESP32 access point; it is not a traffic field-validation result.
ESP32 endpoint latency from repeated log	/status: mean 48.433 ms, p50 54 ms, p95 71 ms, min 12 ms, max 107 ms. Across all /agent attempts, mean 45.715 ms, p50 33 ms, p95 69 ms, min 16 ms, max 1246 ms, with the maximum corresponding to the single timeout; successful /agent calls had mean 43.441 ms and max 182 ms.	Reports phone-observed HTTP endpoint latency for the repeated connected AP-mode session, not firmware-only execution time.
Repeated phone Java versus ESP32 agreement	Among successful /agent responses, phone and ESP32 outputs matched in 528/528 comparisons for safety state, rider state, and motion gate; no mismatches were observed.	Supports repeated normal/moderate-load phone–ESP32 agreement under the observed session conditions; broader multi-scenario live agreement remains future work.
Phone battery and wearable summary observations	Watch heart-rate summaries ranged from 75.4 to 100.5 bpm; phone battery changed from 78% to 76%; phone-reported current ranged from −678 to −187 μA.	Phone/watch observations only; not direct ESP32-S3 energy evidence.
ESP32-S3 bench power measurement	INA219 at I2C address 0×40 and MPU6500 at 0×68 were detected on the ESP32-S3 I2C bus. A connected power-bank bench check reported approximately 4.90–4.91 V, 148.7–160.0 mA, and 724–740 mW while motion windows remained motion_ok.	Provides a short directly instrumented bench observation for the ESP32-S3 sensor node; it is not a fixed-duration ride energy profile or long-duration deployment-power claim.

**Table 21:** Descriptive instrumentation results from the primary 16.63 min technical ride performed by one participant on 18 July 2026, together with a separate ESP32 bench check. These measurements describe logging, connectivity, telemetry, and implementation agreement; they do not estimate rider-stress detection accuracy or generalizability.

Automatic Evidence Block	Rows/Duration	Auto-Derived Condition	Phone-Side Result	ESP32-Side Result	Agreement/Quality	Energy/GPS Result
ESP32 bench check	8 s serial sample	ESP32-S3 + MPU6500 + INA219 powered through power bank	Phone not used for this bench block	motion_ok; INA219 good	ESP32 sensors detected and telemetry produced	4.90–4.91 V; 148.7–160.0 mA; 724–740 mW
Ride coverage	963 rows; 16.63 min	Real technical ride pilot; GPS fill 100.0%; ESP32 connected 99.0%	759 normal; 204 watch	763 normal; 193 watch; 3 motion-compromised watch; 4 unavailable	221/963 total matches (22.9%); 738 disagreements	4.86 V mean; −160.3 mA mean; 743 mW mean; 750 mW p95
Rider load/stress context	963 rows	HR mean 76.6 bpm; HR p95 89 bpm; stress-index mean 0.20, p95 0.35	759 normal-or-low-load; 204 moderate physical load	763 normal-or-low-load; 196 moderate physical load; 4 unavailable	0 rows met the heuristic stress-candidate or possible-false-stress candidate rules; no ground-truth rate is inferred	Power normal in 935 rows; 18 low-voltage flags; 10 power-unavailable rows
GPS terrain	963 rows	268 flat/mild-grade; 356 uphill; 339 downhill; grade p95 15.38%	Terrain logged for post hoc phone-side context analysis	Terrain and power logged for post hoc ESP32 comparison	GPS quality: 907 good; 45 poor-accuracy rejected; 1 jump rejected; 10 usable	Altitude 68.34–102.87 m; speed mean 5.31 m/s, p95 6.62 m/s
Motion reliability	963 rows	Watch accel p95 16.49 m/s^2^; watch gyro p95 1.39 rad/s	Motion gates: 453 OK; 286 watch; 224 compromised	ESP motion gate: 954 compromised; 5 OK; 4 unknown	Main mismatch source: 735 motion-only mismatches	ESP RMS p95 0.68 g; ESP jerk p95 51.35 g/s; reliability mean 0.01
Auto-segment labels	963 rows	954 rough/mount issue; 2 hard accel/brake; 1 smooth road; 6 unknown	App generated ride-segment labels without manual entry	ESP IMU jerk fusion dominated the auto segment output	Auto-segment confidence mean 0.99, p95 1.00	Segment labels saved with GPS, power, and agent fields in the CSV

**Table 22:** Extended empirical privacy audit. These checks support data-minimization and guardrail claims only; they are not differential privacy, information-theoretic secrecy, or a formal non-reconstruction proof. All reconstruction attackers see only the six bounded HR summaries listed in the text.

Privacy Check	Status	Result	Safe Interpretation
Direct attack blocking	COMPLETE	31/31 attack payloads blocked; attack blocking rate 1.000	Tested guard patterns were rejected, including raw streams, identifiers, encoded signal, nested raw fields, and long arrays; aliases outside the tested set remain a residual risk.
Generated-output term scan	COMPLETE	Forbidden terms appear mainly in privacy-test fixture reports and manuscript/audit text; the scan is conservative and does not by itself prove payload leakage.	Term hits require context review; attack reports necessarily contain names of rejected fields.
Attribute inference from bounded WESAD summaries	COMPLETE	Random forest LOSO: accuracy 0.896, balanced accuracy 0.883, macro-F1 0.885; class-prior baseline 0.646.	Bounded features can still disclose intended stress-condition evidence; this limits privacy claims.
Raw-signal reconstruction, constrained optimization	COMPLETE	120 pooled windows, no subject-wise split: median RMSE 0.958 bpm, median MAE 0.927 bpm, 51.7% under 1 bpm RMSE.	Bounded summaries are not raw streams, but short-window summaries can still carry reconstructive signal; this limits privacy claims.
Raw-signal reconstruction, constant/mean baseline	COMPLETE	Subject-wise split (8 held-out subjects, 400 windows): median RMSE 0.986 bpm, median MAE 0.942 bpm, 50.5% under 1 bpm RMSE.	No-training baseline; sets the floor that learned attackers must beat.
Raw-signal reconstruction, nearest-neighbour	COMPLETE	Median RMSE 0.779 bpm, median MAE 0.707 bpm, 58.0% under 1 bpm RMSE.	Reusing a training-library sequence with a similar summary already beats the constant baseline.
Raw-signal reconstruction, ridge regression	COMPLETE	Median RMSE 0.638 bpm, median MAE 0.574 bpm, 67.3% under 1 bpm RMSE.	A simple linear learned attacker improves on the constant baseline within the same 400-window subject-wise evaluation.
Raw-signal reconstruction, random forest/XGBoost	COMPLETE	Random forest: median RMSE 0.590 bpm, 71.5% under 1 bpm RMSE. XGBoost: median RMSE 0.628 bpm, 67.5% under 1 bpm RMSE.	These are the strongest results within the common 400-window subject-wise evaluation; they are not directly ranked against the separate 120-window optimization test.
Many-to-one summary demonstration	COMPLETE	Two distinct synthetic 60-sample HR sequences map to identical mean, standard deviation, range, and drift summaries while differing by 9.33 bpm mean absolute sample distance.	Exact unique inversion is not determined by that bounded summary alone; this is structural evidence, not a formal proof.

## Data Availability

Code, trained models, derived evaluation data, and archived results supporting this study are openly available in the OSF repository at https://doi.org/10.17605/OSF.IO/Z7PEB. The original PAMAP2, WESAD, PhysioNet, and GiroE datasets are third-party public datasets available from their respective official repositories (cited in the manuscript) and are not redistributed. Raw GPS- and heart-rate-bearing ride logs from the two author self-testing rides are not publicly released because they contain location and physiological information about the author-rider; aggregate outputs derived from these rides are included in the OSF record. The new revision-analysis scripts and outputs are also supplied in the revised reproducibility archive accompanying this resubmission; that archive is prepared to replace the current OSF file.

## Abbreviations

The following abbreviations are used in this manuscript:

AI	Artificial intelligence
AUPRC	Area under the precision–recall curve
AUROC	Area under the receiver operating characteristic curve
BVP	Blood-volume pulse
EDA	Electrodermal activity
FSER	Exercise false-stress rate
GDPR	General Data Protection Regulation
GNSS	Global navigation satellite system
GPS	Global positioning system
HR	Heart rate
IMU	Inertial measurement unit
IoT	Internet of Things
LOSO	Leave-one-subject-out
MCC	Matthews correlation coefficient
PPG	Photoplethysmography
RQ	Research question

## References

[1] Teschke K., Harris M.A., Reynolds C.C.O., Winters M., Babul S., Chipman M., Cusimano M.D., Brubacher J.R., Hunte G., Friedman S.M. (2012). Route Infrastructure and the Risk of Injuries to Bicyclists: A Case-Crossover Study. Am. J. Public Health.

[2] Dozza M., Bianchi Piccinini G.F., Werneke J. (2016). Using Naturalistic Data to Assess e-Cyclist Behavior. Transp. Res. Part F Traffic Psychol. Behav..

[3] Schleinitz K., Petzoldt T., Franke-Bartholdt L., Krems J., Gehlert T. (2017). The German Naturalistic Cycling Study: Comparing Cycling Speed of Riders of Different e-Bikes and Conventional Bicycles. Saf. Sci..

[4] Cherry C., Cervero R. (2007). Use Characteristics and Mode Choice Behavior of Electric Bike Users in China. Transp. Policy.

[5] Fishman E. (2016). Bikeshare: A Review of Recent Literature. Transp. Rev..

[6] Healey J.A., Picard R.W. (2005). Detecting Stress During Real-World Driving Tasks Using Physiological Sensors. IEEE Trans. Intell. Transp. Syst..

[7] Can Y.S., Arnrich B., Ersoy C. (2019). Stress Detection in Daily Life Scenarios Using Smart Phones and Wearable Sensors: A Survey. J. Biomed. Inform..

[8] Picard R.W., Vyzas E., Healey J. (2001). Toward Machine Emotional Intelligence: Analysis of Affective Physiological State. IEEE Trans. Pattern Anal. Mach. Intell..

[9] Setz C., Arnrich B., Schumm J., La Marca R., Troster G., Ehlert U. (2010). Discriminating Stress from Cognitive Load Using a Wearable EDA Device. IEEE Trans. Inf. Technol. Biomed..

[10] Allen J. (2007). Photoplethysmography and its Application in Clinical Physiological Measurement. Physiol. Meas..

[11] Tamura T., Maeda Y., Sekine M., Yoshida M. (2014). Wearable Photoplethysmographic Sensors—Past and Present. Electronics.

[12] Bent B., Goldstein B.A., Kibbe W.A., Dunn J.P. (2020). Investigating Sources of Inaccuracy in Wearable Optical Heart Rate Sensors. npj Digit. Med..

[13] European Parliament and Council of the European Union (2016). Regulation (EU) 2016/679: General Data Protection Regulation. https://eur-lex.europa.eu/eli/reg/2016/679/oj.

[14] Parasuraman R., Sheridan T.B., Wickens C.D. (2000). A Model for Types and Levels of Human Interaction with Automation. IEEE Trans. Syst. Man Cybern.-Part A Syst. Hum..

[15] Parasuraman R., Manzey D.H. (2010). Complacency and Bias in Human Use of Automation: An Attentional Integration. Hum. Factors.

[16] Endsley M.R. (1995). Toward a Theory of Situation Awareness in Dynamic Systems. Hum. Factors.

[17] Endsley M.R. (2017). From Here to Autonomy: Lessons Learned From Human–Automation Research. Hum. Factors.

[18] Lee J.D., See K.A. (2004). Trust in Automation: Designing for Appropriate Reliance. Hum. Factors.

[19] Goddard K., Roudsari A., Wyatt J.C. (2012). Automation Bias: A Systematic Review of Frequency, Effect Mediators, and Mitigators. J. Am. Med. Inform. Assoc..

[20] Kaya O., Athanassopoulou N., Malliaras G.G., Alban-Paccha M.V. (2026). Differentiating Physical and Psychological Stress Using Wearable Physiological Signals and Salivary Cortisol. arXiv.

[21] Abdel-Latif M., Askari M.R., Rashid M.M., Park M., Sharp L., Quinn L., Cinar A. (2023). Multi-Task Classification of Physical Activity and Acute Psychological Stress for Advanced Diabetes Treatment. Signals.

[22] Reiss A., Stricker D. (2012). PAMAP2 Physical Activity Monitoring.

[23] Schmidt P., Reiss A., Duerichen R., Marberger C., Van Laerhoven K. Introducing WESAD, a Multimodal Dataset for Wearable Stress and Affect Detection. Proceedings of the 20th ACM International Conference on Multimodal Interaction (ICMI ’18).

[24] Hongn A., Bosch F., Prado L., Bonomini P. (2025). Wearable Device Dataset from Induced Stress and Structured Exercise Sessions. PhysioNet, Version 1.0.1. https://physionet.org/content/wearable-device-dataset/1.0.1/.

[25] Hongn A., Bosch F., Prado L.E., Ferrández J.M., Bonomini M.P. (2025). Wearable Physiological Signals under Acute Stress and Exercise Conditions. Sci. Data.

[26] Peffers K., Tuunanen T., Rothenberger M.A., Chatterjee S. (2007). A Design Science Research Methodology for Information Systems Research. J. Manag. Inf. Syst..

[27] Vasey B., Nagendran M., Campbell B., Clifton D.A., Collins G.S., Denaxas S., Denniston A.K., Faes L., Geerts B., Ibrahim M. (2022). Reporting Guideline for the Early-Stage Clinical Evaluation of Decision Support Systems Driven by Artificial Intelligence: DECIDE-AI. Nat. Med..

[28] Collins G.S., Moons K.G.M., Dhiman P., Riley R.D., Beam A.L., Van Calster B., Ghassemi M., Liu X., Reitsma J.B., van Smeden M. (2024). TRIPOD+AI Statement: Updated Guidance for Reporting Clinical Prediction Models That Use Regression or Machine Learning Methods. BMJ.

[29] Sano A., Picard R.W. Stress Recognition Using Wearable Sensors and Mobile Phones. Proceedings of the 2013 Humaine Association Conference on Affective Computing and Intelligent Interaction.

[30] Hovsepian K., al’Absi M., Ertin E., Kamarck T., Nakajima M., Kumar S. cStress: Towards a Gold Standard for Continuous Stress Assessment in the Mobile Environment. Proceedings of the 2015 ACM International Joint Conference on Pervasive and Ubiquitous Computing (UbiComp ’15).

[31] Gjoreski M., Gjoreski H., Luštrek M., Gams M. Continuous Stress Detection Using a Wrist Device: In Laboratory and Real Life. Proceedings of the 2016 ACM International Joint Conference on Pervasive and Ubiquitous Computing: Adjunct.

[32] Smets E., Casale P., Großekathöfer U., Lamichhane B., De Raedt W., Bogaerts K., Van Diest I., Van Hoof C. (2016). Comparison of Machine Learning Techniques for Psychophysiological Stress Detection. Pervasive Computing Paradigms for Mental Health.

[33] van de Ven S.R.B., Gevonden M.J., Noordzij M.L., de Geus E.J.C. (2025). Accelerometer-Based Heart Rate Adjustment for Ambulatory Stress Research. Psychophysiology.

[34] Reiss A., Stricker D. Introducing a New Benchmarked Dataset for Activity Monitoring. Proceedings of the 2012 16th International Symposium on Wearable Computers.

[35] Moser M.K., Schmidt S., Graf D.R.M., Keskin M., Gandhi S., Yap W., Zeile P., Heinke M., Resch B. (2026). Understanding the Influence of Urban Characteristics on Cyclists’ Stress Measured through Wearable Sensors: A Quantitative Open Data Approach. Environ. Plan. B Urban Anal. City Sci..

[36] Bellagente P., Brandão D., Dello Iacono S., Ferrari P., Flammini A., Gaffurini M., Gaioni L., Malighetti P., Rinaldi S., Sisinni E. (2026). Smartwatch Data from Non-Competitive Athletes during the GiroE-2024 E-Bike Multi-Stage Race. Data Brief.

[37] Pejhan S., Agelin-Chaab M., Yusuf M., Eng D. (2021). Analysis of Ebike Dynamics and Cyclists’ Anxiety Levels and Interactions with Road Vehicles That Influence Safety. Accid. Anal. Prev..

[38] Su S., Fidler M., Palmberg R., Susilo Y.O. (2026). Understanding the Physiological Stress of E-Scooter Riders in a Shared Bike Lane: A Study through Virtual Reality. Transp. Plan. Technol..

[39] Su S., Stark J., Fidler M., Hössinger R., Susilo Y.O. (2025). Exploring Physiological Stress of Travelling by Bicycle and e-Scooter in Bicycle Lane: A Comparison Study through Virtual Reality. Travel Behav. Soc..

[40] Shah S.T.A., Santos J.P., Pereira A.B., Constantinescu G., Fernandes J.M. Urban Biometrics in Motion: A Longitudinal Sensor Fusion Framework for Quantifying Traffic-Induced Stress in Micromobility Environments. Proceedings of the 2026 12th International Conference on Computing and Data Engineering.

[41] Kegalle H.N., Hettiachchi D., Chan J., Sanderson M., Salim F.D. (2025). Watch Out! E-scooter Coming Through!: Multimodal Sensing of Mixed Traffic Use and Conflicts Through Riders’ Ego-centric Views. Proceedings of the ACM on Interactive, Mobile, Wearable and Ubiquitous Technologies.

[42] Shah S.T.A., Fernandes J.M., Santos J.P., Constantinescu G., Pereira A.B. (2026). The Micro-Mobility Sensing Gap: A Systematic Review of Physiological Safety Monitoring from Cycling to E-Scooters. Sensors.

[43] Lara O.D., Labrador M.A. (2013). A Survey on Human Activity Recognition using Wearable Sensors. IEEE Commun. Surv. Tutor..

[44] Bulling A., Blanke U., Schiele B. (2014). A Tutorial on Human Activity Recognition Using Body-Worn Inertial Sensors. ACM Comput. Surv..

[45] Kwapisz J.R., Weiss G.M., Moore S.A. (2011). Activity Recognition Using Cell Phone Accelerometers. ACM SIGKDD Explor. Newsl..

[46] Banos O., Galvez J.M., Damas M., Pomares H., Rojas I. (2014). Window Size Impact in Human Activity Recognition. Sensors.

[47] Ordóñez F.J., Roggen D. (2016). Deep Convolutional and LSTM Recurrent Neural Networks for Multimodal Wearable Activity Recognition. Sensors.

[48] Hammerla N.Y., Halloran S., Ploetz T. Deep, Convolutional, and Recurrent Models for Human Activity Recognition Using Wearables. Proceedings of the Twenty-Fifth International Joint Conference on Artificial Intelligence.

[49] Dehghani A., Glatard T., Shihab E. (2019). Subject Cross Validation in Human Activity Recognition. arXiv.

[50] Wang J., Chen Y., Hao S., Peng X., Hu L. (2019). Deep Learning for Sensor-based Activity Recognition: A Survey. Pattern Recognit. Lett..

[51] Elgendi M. (2012). On the Analysis of Fingertip Photoplethysmogram Signals. Curr. Cardiol. Rev..

[52] Castaneda D., Esparza A., Ghamari M., Soltanpur C., Nazeran H. (2018). A Review on Wearable Photoplethysmography Sensors and Their Potential Future Applications in Health Care. Int. J. Biosens. Bioelectron..

[53] Charlton P.H., Allen J., Bailón R., Baker S., Behar J.A., Chen F., Clifford G.D., Clifton D.A., Davies H.J., Ding C. (2023). The 2023 Wearable Photoplethysmography Roadmap. Physiol. Meas..

[54] Shcherbina A., Mattsson C.M., Waggott D., Salisbury H., Christle J.W., Hastie T., Wheeler M.T., Ashley E.A. (2017). Accuracy in Wrist-Worn, Sensor-Based Measurements of Heart Rate and Energy Expenditure in a Diverse Cohort. J. Pers. Med..

[55] Gillinov S., Etiwy M., Wang R., Blackburn G., Phelan D., Gillinov A.M., Houghtaling P., Javadikasgari H., Desai M.Y. (2017). Variable Accuracy of Wearable Heart Rate Monitors During Aerobic Exercise. Med. Sci. Sport. Exerc..

[56] Stahl S.E., An H.S., Dinkel D.M., Noble J.M., Lee J.M. (2016). How Accurate Are the Wrist-Based Heart Rate Monitors During Walking and Running Activities? Are They Accurate Enough?. BMJ Open Sport Exerc. Med..

[57] Zhang Z., Pi Z., Liu B. (2015). TROIKA: A General Framework for Heart Rate Monitoring Using Wrist-Type Photoplethysmographic Signals During Intensive Physical Exercise. IEEE Trans. Biomed. Eng..

[58] Zhang Z. (2015). Photoplethysmography-Based Heart Rate Monitoring in Physical Activities via Joint Sparse Spectrum Reconstruction. IEEE Trans. Biomed. Eng..

[59] Pérez-Zuriaga A.M., Llopis-Castelló D., Just-Martínez V., Fonseca-Cabrera A.S., Alonso-Troyano C., García A. (2022). Implementation of a Low-Cost Data Acquisition System on an E-Scooter for Micromobility Research. Sensors.

[60] David R., Duke J., Jain A., Janapa Reddi V., Jeffries N., Li J., Kreeger N., Nappier I., Natraj M., Wang T. (2021). TensorFlow Lite Micro: Embedded Machine Learning for TinyML Systems. Proc. Mach. Learn. Syst..

[61] Kallimani R., Pai K., Raghuwanshi P., Iyer S., López O.L.A. (2024). TinyML: Tools, Applications, Challenges, and Future Research Directions. Multimed. Tools Appl..

[62] Ganesan P., Thota Y.R., Shehata H., Nikoubin T. TinyML Based Stress Detection utilizing PPG Signals: A Lightweight Approach for Smart Wearable Devices. Proceedings of the Great Lakes Symposium on VLSI 2025.

[63] Abu-Samah A., Ghaffar D., Abdullah N.F., Kamal N., Nordin R., Dela Cruz J.C., Magwili G.V., Mercado R.J. (2025). Deployment of TinyML-Based Stress Classification Using Computational Constrained Health Wearable. Electronics.

[64] Shi W., Cao J., Zhang Q., Li Y., Xu L. (2016). Edge Computing: Vision and Challenges. IEEE Internet Things J..

[65] Merenda M., Porcaro C., Iero D. (2020). Edge Machine Learning for AI-Enabled IoT Devices: A Review. Sensors.

[66] Mendez J., Bierzynski K., Cuellar M.P., Morales D.P. (2022). Edge Intelligence: Concepts, Architectures, Applications, and Future Directions. ACM Trans. Embed. Comput. Syst..

[67] Singh R., Gill S.S. (2023). Edge AI: A Survey. Internet Things-Cyber-Phys. Syst..

[68] Reddi V.J., Cheng C., Kanter D., Mattson P., Schmuelling G., Wu C.J., Anderson B., Breughe M., Charlebois M., Chou W. MLPerf Inference Benchmark. Proceedings of the 2020 ACM/IEEE 47th Annual International Symposium on Computer Architecture (ISCA).

[69] Sze V., Chen Y.H., Yang T.J., Emer J.S. (2017). Efficient Processing of Deep Neural Networks: A Tutorial and Survey. Proc. IEEE.

[70] Dwork C., McSherry F., Nissim K., Smith A. Calibrating Noise to Sensitivity in Private Data Analysis. Proceedings of the Third Theory of Cryptography Conference (TCC 2006).

[71] Abadi M., Chu A., Goodfellow I., McMahan H.B., Mironov I., Talwar K., Zhang L. Deep Learning with Differential Privacy. Proceedings of the 2016 ACM SIGSAC Conference on Computer and Communications Security.

[72] McMahan B., Moore E., Ramage D., Hampson S., Aguera y Arcas B. Communication-Efficient Learning of Deep Networks from Decentralized Data. Proceedings of the 20th International Conference on Artificial Intelligence and Statistics.

[73] Kairouz P., McMahan H.B., Avent B., Bellet A., Bennis M., Bhagoji A.N., Bonawitz K., Charles Z., Cormode G., Cummings R. (2021). Advances and Open Problems in Federated Learning. Found. Trends Mach. Learn..

[74] Fredrikson M., Jha S., Ristenpart T. Model Inversion Attacks That Exploit Confidence Information and Basic Countermeasures. Proceedings of the 22nd ACM SIGSAC Conference on Computer and Communications Security.

[75] Shokri R., Stronati M., Song C., Shmatikov V. Membership Inference Attacks Against Machine Learning Models. Proceedings of the 2017 IEEE Symposium on Security and Privacy (SP).

[76] Malekzadeh M., Clegg R.G., Cavallaro A., Haddadi H. Protecting Sensory Data against Sensitive Inferences. Proceedings of the 1st Workshop on Privacy by Design in Distributed Systems.

[77] Amershi S., Weld D., Vorvoreanu M., Fourney A., Nushi B., Collisson P., Suh J., Iqbal S., Bennett P.N., Inkpen K. Guidelines for Human-AI Interaction. Proceedings of the 2019 CHI Conference on Human Factors in Computing Systems.

[78] Hendrickx K., Perini L., Van der Plas D., Meert W., Davis J. (2024). Machine Learning with a Reject Option: A Survey. Mach. Learn..

[79] Dietterich T.G. (1998). Approximate Statistical Tests for Comparing Supervised Classification Learning Algorithms. Neural Comput..

[80] Varoquaux G. (2018). Cross-Validation Failure: Small Sample Sizes Lead to Large Error Bars. NeuroImage.

[81] Saito T., Rehmsmeier M. (2015). The Precision-Recall Plot Is More Informative than the ROC Plot When Evaluating Binary Classifiers on Imbalanced Datasets. PLoS ONE.

[82] Chicco D., Jurman G. (2020). The Advantages of the Matthews Correlation Coefficient (MCC) over F1 Score and Accuracy in Binary Classification Evaluation. BMC Genom..

[83] Daza E.J. (2019). Person as Population: A Longitudinal View of Single-Subject Causal Inference for Analyzing Self-Tracked Health Data. arXiv.

[84] von Elm E., Altman D.G., Egger M., Pocock S.J., Gøtzsche P.C., Vandenbroucke J.P. (2007). The Strengthening the Reporting of Observational Studies in Epidemiology (STROBE) Statement: Guidelines for Reporting Observational Studies. PLoS Med..

